# IL6 suppresses vaccine responses in neonates by enhancing IL2 activity on T follicular helper cells

**DOI:** 10.1038/s41541-023-00764-1

**Published:** 2023-11-08

**Authors:** Swetha Parvathaneni, Jiyeon Yang, Leda Lotspeich-Cole, Jiro Sakai, Robert C. Lee, Mustafa Akkoyunlu

**Affiliations:** US FDA/CBER/OVRR/DBPAP, 10903 New Hampshire Ave., Silver Spring, MD USA

**Keywords:** Lymphocyte differentiation, Conjugate vaccines

## Abstract

The inability of neonates to develop CD4^+^FoxP3^-^CXCR5^hi^PD-1^hi^ T follicular helper (T_FH_) cells contributes to their weak vaccine responses. In previous studies, we measured diminished IgG responses when IL-6 was co-injected with a pneumococcal conjugate vaccine (PCV) in neonatal mice. This is in sharp contrast to adults, where IL-6 improves vaccine responses by downregulating the expression of IL-2Rβ on T_FH_ cells and protecting them from the inhibitory effect of IL-2. In this study, we found that splenic IL-6 levels rapidly increased in both adult and neonatal mice following immunization, but the increase in neonatal mice was significantly more than that of adult mice. Moreover, immunized neonatal T_FH_ cells expressed significantly more IL-2 as well as its receptors, IL-2Rα and IL-2Rβ, than the adult cells. Remarkably, IL-6 co-injection with PCV vaccine further increased the production of IL-2 and the expression of its receptors by neonatal T_FH_ cells, whereas excess IL-6 had totally opposite effect in immunized adult mice. Underscoring the role of IL-6 in activating the IL-2 mediated suppression of vaccine responses, immunization of IL-6 knock-out neonates led to improved antibody responses accompanied by expanded T_FH_ cells as well as lower levels of IL-2 and IL-2 receptors on T_FH_ cells. Moreover, CpG containing PCV improved T_FH_ response in neonates by suppressing the expression of IL-2 receptors on T_FH_ cells and inhibiting IL-2 activity. These findings unveil age-specific differences in IL-6 mediated vaccine responses and highlight the need to consider age-related immunobiological attributes in designing vaccines.

## Introduction

Newborns and infants are vulnerable to infections mostly because their immune system is not as efficient as the adult immune system in controlling microbial assaults and responding to vaccines^1–6^. Incomplete understanding of the underlying mechanisms of suboptimal immune responses to vaccines during early age is an obstacle in improving pediatric vaccines^7^. Studies comparing neonatal and adult immune system have identified phenotypic and functional differences in the innate^8,9^ and adaptive arms^10–14^ of the immune system between the age groups. In adult mice, generation of protective antibody responses to T-cell-dependent vaccines relies on optimum germinal center (GC) reaction in secondary lymphoid organs^15^. The GC response involves the development of antigen specific T follicular helper (T_FH_) cells expressing the chemokine receptor CXCR5 as well as PD-1 which then migrate into B cell follicles^15–17^. T_FH_ cells become fully committed with the expression of the transcription factor Bcl6 in response to cytokines IL-6 and IL-21. Activated T_FH_ cells interact with GC B cells which then differentiate into antibody secreting plasma cells or memory B cells^16,18^. Tight regulation by cells and molecules that promote or inhibit the GC reaction is needed to facilitate the development of high affinity antibodies against pathogens^19^. For example, FoxP3-expressing follicular regulatory T (T_FR_) cells in the GC limit T_FH_ and GC B cell responses to terminate the GC reaction^20^ and also prevent autoreactive and allergen specific antibody production^21–23^. Counterbalancing the T_FH_ promoting activity of IL-21 and IL-6, cytokines IL-2 and IL-7 limit T_FH_ expansion through STAT5 activation^24,25^.

In neonates, the insufficient antibody responses to vaccines are associated with inadequate expansion of T_FH_ cells and GC B cells^13,14,26–28^. The underlying reasons for this blunted GC response are poorly understood. Our previous research indicated a predominance of T_FR_ cells in vaccinated neonatal mice^14^, which could indicate dampened GC response^23^. Importantly, we found that IL-6 co-injection suppressed neonatal T_FH_ generation and diminished antibody responses^14^. In adult mice, IL-6 protects T_FH_ cells from the well-established IL-2 mediated inhibition^24^ by downregulating the expression of IL-2Rβ (CD122) on T_FH_ cells and limiting IL-2 induced signaling^29^.

Here, we sought to assess how IL-6 regulates IL-2 activity on T_FH_ cells in immunized neonatal mice. We found that not only did immunized neonatal mice T_FH_ cells produce more IL-6 and IL-2 than those in adults, but they also expressed higher IL-2Rα and IL-2Rβ. Experiments performed in IL-6 co-injected wild-type neonatal and IL-6 deficient (IL-6 KO) neonatal mice indicated that, contrary to its IL-2Rβ suppressing effect in adult T_FH_ cells^29^, IL-6 increased IL-2 production by T_FH_ cells and upregulated the expression of IL-2 receptors on neonatal T_FH_ cells. Moreover, immunization of neonatal mice with a CpG containing vaccine improved T_FH_ response which was accompanied by suppressed IL-6 and IL-2 production in addition to downregulated expression of IL-2 receptors on T_FH_ cells. These results provide insight into IL-6-mediated suppression of vaccine responses in neonatal mice.

## Results

### Enhanced IL-6 production and IL-2 activity in neonatal T_FH_ cells following immunization

**Fig. 1:**
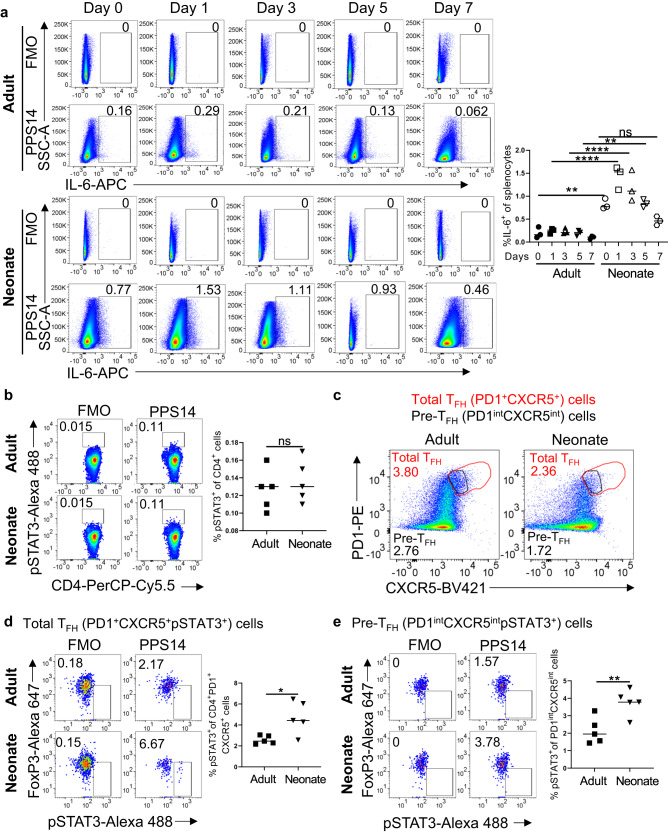
Germinal center response to PPS14-TT vaccination in neonatal and adult mice. Adult and neonatal mice were immunized with PPS14-TT and splenocytes were analyzed by FACS. **a** Representative dot plots from 0, 1, 3, 5, and 7 dpi depict the fluorescence minus one (FMO) controls and the percentages of IL-6^+^ cells gated on total splenocytes. Mean percentages of IL-6^+^ cells among splenocytes are plotted (*n* = 3). FMOs for each age group are the same for Day 1 and Day 5 because the samples for these days were analyzed on the same day. Also, FMOs for adult and neonates are the same for Day 3 because cells from adult and neonatal mice were pooled due to insufficient number of cells for each age group on this time point. Experiment was performed two times. **b–e** Adult and neonatal mice were immunized i.p. with PPS14-TT and 24 h post immunization (hpi) splenocytes were analyzed for p-STAT3 levels by FACS. **b** Representative FACS plots from 24 hpi splenocytes depict the percentages of p-STAT3^+^ cells on total CD4^+^ cells from adult and neonatal mice. Mean percentages of p-STAT3^+^ cells among total CD4^+^ cells are plotted (*n* = 5). **c** Pre-gated CD4^+^ cells were further gated for total T_FH_ (PD-1^+^CXCR5^+^) and pre-T_FH_ (PD-1^int^CXCR5^int^) populations in adult and neonatal mice. **d** The percentages of total T_FH_ (PD-1^+^CXCR5^+^) cells expressing FoxP3^−^p-STAT3^+^ were analyzed. Representative FACS plots depict the percentages of FoxP3^−^p-STAT3^+^ cells on total T_FH_ cells. Mean percentages of FoxP3^−^p-STAT3^+^ cells among total T_FH_ cells are plotted (*n* = 5). **e** The percentages of PD-1^int^CXCR5^int^ cells expressing FoxP3^−^p-STAT3^+^ were analyzed. Representative FACS plots depict the percentages of FoxP3^-^p-STAT3^+^ cells on PD-1^int^CXCR5^int^ cells. Mean percentages of FoxP3^−^p-STAT3^+^ cells among CD4^+^PD-1^int^CXCR5^int^ cells are plotted (*n* = 5). Experiment was performed three times. Unpaired student’s *t*-test and One-Way ANOVA were used for all comparisons; data represented as mean ± SEM are shown. *P* values < 0.05 were considered statistically significant. *P < 0.05, **P < 0.01, ****P < 0.0001 and ns (non-significant).

We have previously shown that IL-6 co-injection suppresses neonatal vaccine responses^14^. In adult mice, IL-6 plays an important role in the improved host response to vaccines^29–32^, and adjuvanted vaccines elicit peak IL-6 production in lymphoid organs during the first 24 h after vaccination^33^. To assess the regulation of IL-6 production in neonatal splenic cells following vaccination, we immunized adult (6- to 10-week-old) and neonatal (5- to 7-day-old) mice with aluminum hydroxide-adjuvanted tetanus toxoid-conjugated pneumococcal type 14 serotype (PPS14-TT) vaccine. As expected, immunization induced significantly higher frequency of CD4^+^CXCR5^hi^PD-1^hi^ FoxP3^−^ T_FH_ cells in adults than in neonates (Supplementary Fig. S1a, b). As shown previously^33^, in adult mice the frequency of IL-6-expressing splenocytes slightly increased 24 h after immunization and gradually decreased thereafter (Fig. 1a and Supplementary Fig. 1c). The kinetics of splenic IL-6 expression in neonatal splenocytes overlapped with that of adult mice. However, the frequency of splenocytes expressing IL-6 in immunized neonatal mice was much higher than those of adult mice during the first 5 days after immunization. We observed a similar kinetics in the frequency of IL-6-expressing CD11c^+^, F4/80^+^, CD19^+^ and CD3^+^ cells as the total splenic cells for both the age groups (Supplementary Figs. 1c, 2a, b, and 3a, b). Importantly, IL-6 expression was significantly higher in neonates compared to adults for all these cells. To test whether the increase in IL-6 production in neonatal splenic cells translated into in vivo phosphorylation of STAT3 (p-STAT3) in CD4^+^ cells^29^, we examined p-STAT3^+^ populations in adult and neonatal mice spleens 24 h after immunization, the peak time point of IL-6 production (Fig. 1a). We detected no differences in the frequencies of adult and neonatal CD4^+^p-STAT3^+^ cell populations (Fig. 1b and Supplementary Fig. 4a). Similarly, the frequencies of FoxP3^−^p-STAT3^+^ cells among the total CD4^+^ cells were comparable between the two age groups (Supplementary Fig. 4c, d). Next, we assessed p-STAT3^+^ population after further gating of CD4^+^ cells for total T_FH_ cells which included both the CD4^+^FoxP3^−^CXCR5^int^PD-1^int^ pre-T_FH_ cells and CD4^+^FoxP3^−^CXCR5^hi^PD-1^hi^ genuine T_FH_ population. We drew the T_FH_ gate larger to include pre-T_FH_ population also because majority of the early T_FH_ cells are composed of pre-T_FH_ during the first three days of immunization (Fig. 1c and Supplementary Fig. 4b)^34^. We found that both the total T_FH_ (Fig. 1d) and pre-T_FH_ (Fig. 1e) cells included more p-STAT3^+^ population in neonates than in adults. We also attempted to measure the p-STAT3^+^ population among the CD4^+^FoxP3^−^CXCR5^hi^PD-1^hi^ cells but the low cell count prevented reproducible measurement of p-STAT3^+^ cells. Nevertheless, higher percentage of p-STAT3^+^ cells among the pre- T_FH_ and total T_FH_ populations in neonatal mice suggests that the increased IL-6 produced by neonatal splenic cells is likely responsible for the difference in p-STAT3^+^ T_FH_ cell populations between neonatal and adult mice.

**Fig. 2:**
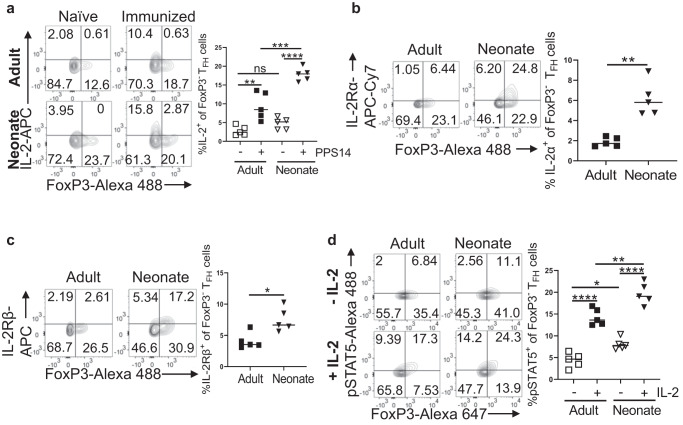
Neonatal mice have higher IL-2 levels and IL-2 receptors than the adult mice. **a** Adult and neonatal mice were immunized with PPS14-TT and splenocytes were analyzed for IL-2 expression 7 dpi by FACS. Representative contour plots depict the percentages of IL-2-expressing FoxP3^+^ and FoxP3^−^ cells on T_FH_ cells. Mean percentages of IL-2^+^ cells among T_FH_ cells are plotted (*n* = 5). **b**, **c** Splenocytes were analyzed for IL-2 receptor expression 7 dpi by FACS. Representative contour plots depict percentages of IL-2Rα- (**b**) or IL-2Rβ- (**c**) expressing FoxP3^+^ and FoxP3^−^ cells on T_FH_ cells. Mean percentages of IL-2Rα^+^ and IL-2Rβ^+^ cells among T_FH_ cells are also plotted (*n* = 5). **d** Splenocytes from 7 dpi were stimulated with or without recombinant IL-2 for 15 min, followed by intracellular staining for p-STAT5. Representative contour plots depict the percentages of p-STAT5^+^ cells among FoxP3^+^ and FoxP3^−^ T_FH_ (pre-gated on CD4^+^CXCR5^hi^PD-1^hi^) cells. Mean percentages of p-STAT5^+^ cells among T_FH_ cells are plotted (*n* = 5). The data are representative of at least two independent experiments. Each experiment was performed twice. Unpaired student’s t-test and One-Way ANOVA were used for all comparisons; data represented as mean ± SEM are shown. P values < 0.05 were considered statistically significant. *P < 0.05, **P < 0.01, ***P < 0.001, ****P < 0.0001 and ns (non-significant).

Next, we focused on IL-2 production in immunized mice because a recent report demonstrated that CD4^+^FoxP3^−^ T cells spontaneously produced significantly more IL-2 in unimmunized neonatal mice compared to adults and suggested that the elevated IL-2 production likely contributes to the ablated T_FH_ development in immunized neonatal mice^28^. Consistent with this report, we found higher IL-2 expression by freshly isolated naive neonatal CD4^+^ T cells than the naive adult counterparts (Supplementary Fig. 5a, b). The proportion of neonatal CD4^+^IL-2^+^ cells compared to those of adults increased further 7 days after immunization as assessed by ex vivo staining (Supplementary Fig. 5b) or following phorbol myristate acetate/ionomycin (PMA/Ion) stimulation (Supplementary Fig. 5c). We also measured the frequencies of IL-2^+^ T_FH_ cells in adult and neonatal mice because Papillon and colleagues showed that the expression of IL-2 by T_FH_ cells is more relevant for T_FH_ inhibition than IL-2 produced by other cells^29^. As with total CD4^+^ cells, naive neonatal T_FH_ cells expressed more IL-2 than their adult counterparts and this difference increased further after immunization when measured ex vivo (Fig. 2a and Supplementary Fig. 5d). The same phenomenon was observed following PMA/Ion stimulation (Supplementary Fig. 5e). While PMA/Ion stimulation means only a subset of T_FH_ cells are captured, the proportion of T_FH_ cells that secrete IL-2 was higher in neonatal cells compared to adult cells. The increase in IL-2 production by T_FH_ cells following PMA/Ion stimulation of cells was meaningful because there was a significant decrease in the frequency of neonatal and adult T_FH_ cells proportional to the increase in IL-2 production (Supplementary Fig. 5f). The decrease in T_FH_ cell population is likely mediated by the increase in IL-2 production since excess IL-2 effectively inhibits CXCR5 and PD-1 expression^24^ and conversely, inhibition of IL-2 leads to increase in CXCR5 and PD-1 expressing T_FH_ cells^28^ through the suppression of Bcl6^35–37^.

In addition to the amount of IL-2 produced by T_FH_ cells, the permissiveness of T_FH_ cells to IL-2 mediated suppression also depends on the level of IL-2 receptor expression by T_FH_ cells^29^, and in adult mice IL-6 alleviates IL-2 mediated T_FH_ inhibition by downregulating IL-2Rβ expression on T_FH_ cells^29^. We therefore compared IL-2Rα and IL-2Rβ expression on adult and neonatal T_FH_ cells following immunization. We found significantly higher expression of IL-2Rα and IL-2Rβ on neonatal T_FH_ cells compared to adult cells (Fig. 2b, c and Supplementary Fig. 6a). Thus, not only did neonatal T_FH_ cells produce more IL-2 than the adult cells, but also, they expressed higher levels of IL-2 receptors than adult T_FH_ cells.

The T_FH_ inhibitory activity of IL-2 is mediated by STAT5^38^. To test whether elevated IL-2Rα and IL-2Rβ resulted in enhanced STAT5 activation in T_FH_ cells, we isolated splenocytes seven days post immunization (dpi) and subjected them to IL-2 stimulation. We found that even unstimulated neonatal FoxP3^−^ T_FH_ cells contained significantly higher phospho-STAT5^+^ (p-STAT5^+^) cells than those in adult cells (Fig. 2d and Supplementary Fig. 6b). More importantly, correlating with the increase in IL-2Rα and IL-2Rβ expression, IL-2 stimulation induced significantly higher p-STAT5^+^ T_FH_-cell frequency in immunized neonates than in adults (Fig. 2d). Since IL-2 induced STAT5 activation blunts the expression of *Bcl6*^24,38,39^, diminished T_FH_ development in neonatal mice is likely due to elevated IL-2Rα and IL-2Rβ expression on T_FH_ cells in addition to increased IL-2 production by T_FH_ cells^28^.

### Co-injection of IL-6 suppresses vaccine responses in neonates by enhancing IL-2 activity

**Fig. 3:**
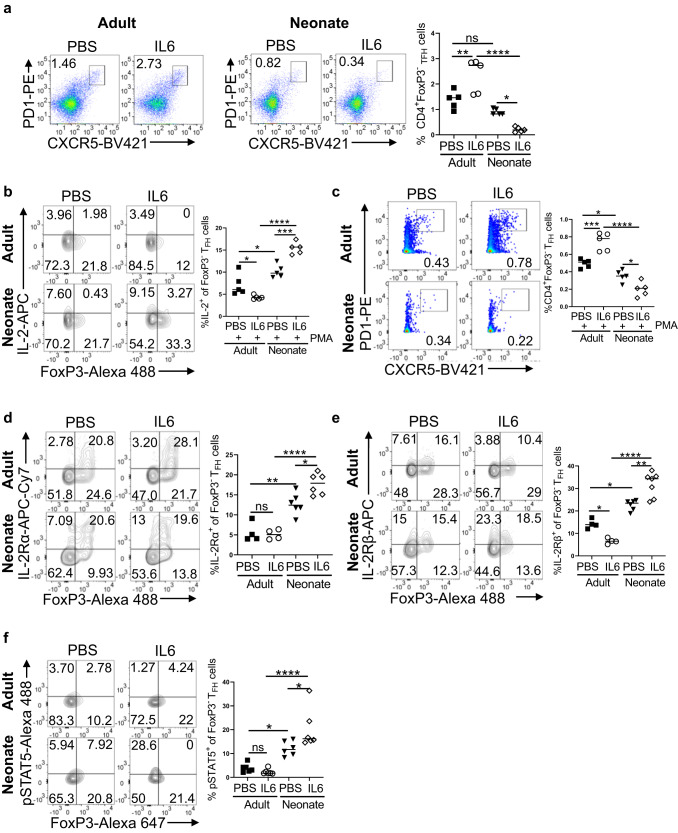
Germinal center response to IL-6 co-injected PPS14-TT vaccine in neonatal and adult mice. Adult and neonatal mice were immunized i.p. with PPS14-TT in PBS (PBS) or PPS14-TT + IL-6 (IL-6) and splenocytes were analyzed by FACS at 7 dpi. **a** Representative dot plots depict the percentages of T_FH_ (CXCR5^hi^PD-1^hi^) cells pre-gated on CD4^+^FoxP3^−^ cells. Mean percentages of T_FH_ cells are plotted (*n* = 5). **b**, **c** Splenocytes from immunized mice were in vitro stimulated with PMA/Ion for 4 h followed by intracellular staining for IL-2 on T_FH_ cells. **b** Representative contour plots depict the percentages of IL-2-expressing FoxP3^+^ and FoxP3^−^ cells on T_FH_ cells. Mean percentages of IL-2^+^ cells among T_FH_ cells are plotted (*n* = 5). **c** Representative dot plots depict the percentage of T_FH_ (CXCR5^hi^PD-1^hi^) cells pre-gated on CD4^+^FoxP3^−^ cells. Mean percentages of T_FH_ cells are plotted (*n* = 5). **d**, **e** Splenocytes from immunized mice were pre-gated on CD4^+^CXCR5^hi^PD-1^hi^ T_FH_ cells. Representative contour plots depict percentages of IL-2Rα- (**d**) or IL-2Rβ- (**e**) expressing FoxP3^+^ and FoxP3^−^ cells on T_FH_ cells. Mean percentages of IL-2Rα^+^ and IL-2Rβ^+^ cells among T_FH_ cells are plotted (*n* = 4–7). **f** Splenocytes from immunized mice were stimulated with recombinant IL-2 for 15 min, followed by intracellular staining for pSTAT5. Representative contour plots depict the percentage of pSTAT5^+^ cells on T_FH_ (CXCR5^hi^PD-1^hi^FoxP3^−^) cells pre-gated on CD4^+^ cells. Mean percentages of p-STAT5^+^ cells among T_FH_ cells are plotted (*n* = 6). Experiments were performed two to four times. One-Way ANOVA was used for all comparisons; data represented as mean ± SEM are shown. *P* values < 0.05 were considered statistically significant. *P < 0.05, **P < 0.01, ***P < 0.001, ****P < 0.0001 and ns (non-significant).

In adult mice, IL-6 enhances vaccine responses by promoting T_FH_ cells^14,30^ through downregulation of IL-2Rβ expression and protecting T_FH_ cells from the inhibitory effect of IL-2^29^. We previously found that IL-6 co-injection with PPS14-TT vaccine suppresses T_FH_ development and antibody responses in neonatal mice^14^. To assess whether IL-6 mediated blunting of PPS14-TT response involves the regulation of IL-2 activity, we co-injected adult and neonatal mice with IL-6 and PPS14-TT vaccine or PPS14-TT vaccine in PBS and analyzed the changes in IL-2 production and the expression of IL-2 receptors on T_FH_ cells. Confirming our previous results^14^, IL-6 co-injection decreased T_FH_ cell frequency in neonatal mice, whereas it increased T_FH_ population in adult mice (Fig. 3a). Also, as we showed before there was a significant increase in FoxP3^+^CD4^+^CXCR5^hi^PD-1^hi^ T_FR_ cells in IL-6 co-injected neonatal mice while adult T_FR_ cells decreased with excess IL-6 (Supplementary Fig. 7a, b). Interestingly, the IL-6-mediated decrease in neonatal T_FH_ cells was accompanied by a reciprocal increase in IL-2 expressing CD4^+^ cells (Supplementary Fig. 7c) as well as FoxP3^−^ T_FH_ cells (Supplementary Fig. 7d) compared to neonates that received PPS14-TT alone. Conversely, IL-6 co-injection with PPS14-TT led to a significant decrease in IL-2 producing CD4+ and FoxP3^−^ T_FH_ cells in adults compared to those injected with PPS14-TT alone. In vitro stimulation of splenocytes from immunized neonatal mice with PMA/Ion further increased the IL-2 production from CD4^+^ (Supplementary Fig. 7e) and T_FH_ cells (Fig. 3b). In parallel to the increase in IL-2^+^ T_FH_ cell population following PMA/Ion stimulation, there was also a statistically significant decrease in the FoxP3^−^ T_FH_ population in IL-6 co-injected neonates compared to those given the vaccine only (Fig. 3c). Moreover, consistent with the previous report^29^, in IL-6 co-injected adult mice there was a decrease in the frequency of IL-2Rβ-expressing (Fig. 3e), but not IL-2Rα-expressing (Fig. 3d), T_FH_ cells compared to those injected with PPS14-TT alone. Likely a consequence of the suppressed IL-2Rβ expression, there was a decrease trend in the frequency of p-STAT5^+^ T_FH_ cells following IL-2 stimulation of splenocytes from IL-6 co-injected adult mice compared to those immunized with PPS14-TT alone (Fig. 3f). In sharp contrast to adult mice, IL-6 co-injection increased both IL-2Rα and IL-2Rβ expression on neonatal T_FH_ cells (Fig. 3d, e). Accompanying the increase in both the IL-2 receptors, IL-6 co-injected neonatal mice T_FH_ cells manifested higher frequency of p-STAT5^+^ population as compared to mice injected with PPS14-TT alone following IL-2 stimulation (Fig. 3f). Thus, the decreased IL-2 production together with the dampened IL-2Rβ expression on T_FH_ cells are likely responsible for the increased T_FH_ cell frequency in IL-6 co-injected adult mice. Paradoxically, in neonates, excess IL-6 further increases the production of IL-2 by T_FH_ cells and stimulates the expression of both the IL-2 receptors on T_FH_ cells, likely rendering them more susceptible to IL-2 mediated suppression.

### IL-6 deficiency improves vaccine responses in neonatal mice

**Fig. 4:**
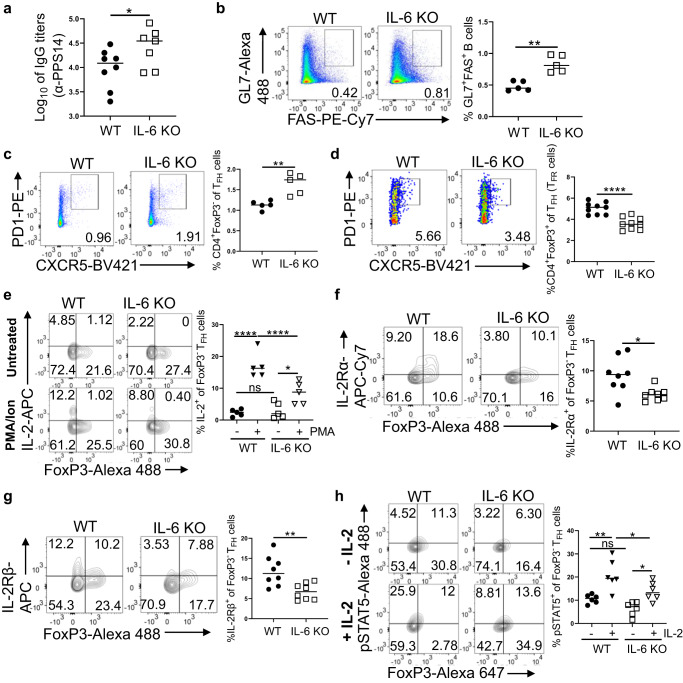
IL-6 KO neonatal mice antibody and germinal center response to PPS14-TT vaccine. Neonatal wild-type (C57BL/6J) and IL-6 KO mice were immunized i.p. with PPS14-TT and splenocytes were analyzed by FACS at 7 dpi. **a** Serum anti-PPS14 IgG titers were determined by ELISA 4 weeks after immunization (n = 8 wild-type and n = 7 IL-6 KO). **b** Representative dot plots depict the GC B (GL7^+^FAS^+^) cells pre-gated on B220^+^ cells. Mean percentages of GC B cells are plotted (n = 5). **c** Representative dot plots depict the GC T_FH_ (CXCR5^hi^PD-1^hi^) cells pre-gated on CD4^+^FoxP3^−^ cells. Mean percentages of FoxP3^−^ T_FH_ cells are plotted (*n* = 5). **d** Representative dot plots depict the GC T_FR_ (CXCR5^hi^PD-1^hi^) cells pre-gated on CD4^+^FoxP3^+^ cells. Mean percentages of T_FR_ cells are plotted (*n* = 9). **e** Splenocytes from immunized mice were in vitro stimulated with PMA/Ion for 4 h and T_FH_ cells were analyzed. Representative contour plots depict the percentages of IL-2-expressing FoxP3^+^ and FoxP3^−^ cells pre-gated on T_FH_ (CD4^+^CXCR5^hi^PD-1^hi^) population. Mean percentages of IL-2^+^ cells among T_FH_ cells are plotted (*n* = 5). **f**, **g** Splenocytes from immunized mice were pre-gated on CD4^+^CXCR5^hi^PD-1^hi^ T_FH_ cells. Representative contour plots depict percentages of IL-2Rα- (**f**) or IL-2Rβ- (**g**) expressing FoxP3^+^ and FoxP3^−^ cells on T_FH_ cells. Mean percentages of IL-2Rα^+^ and IL-2Rβ^+^ cells among T_FH_ cells are also plotted (*n* = 8). **h** Splenocytes from immunized mice were stimulated with or without IL-2 for 15 min, followed by intracellular staining for p-STAT5. Representative contour plots depict the percentages of p-STAT5^+^ cells among FoxP3^+^ and FoxP3^−^ T_FH_ (pre-gated on CD4^+^CXCR5^hi^PD-1^hi^) cells. Mean percentages of p-STAT5^+^ cells among T_FH_ cells are plotted (*n* = 6). Experiments were performed two to seven times. Unpaired student’s *t*-test and One-Way ANOVA were used for all comparisons; data represented as mean ± SEM are shown. *P* values < 0.05 were considered statistically significant. *P < 0.05, **P < 0.01, ****P < 0.0001 and ns (non-significant).

Immunization of neonatal C57BL/6 mice indicated that elevated IL-6 together with increased IL-2 production and upregulated IL-2Rα and IL-2Rβ may be preventing the expansion of T_FH_ cells (Figs. 1 and 2). IL-6 co-injection studies strengthened this hypothesis because IL-6 further induced IL-2 and increased IL-2Rα and IL-2Rβ expression by T_FH_ cells (Fig. 3). Collectively, these data support a role for IL-6 in IL-2 mediated suppression of T_FH_ generation in neonates (Fig. 3a)^14^. To test this hypothesis, we immunized wild-type and IL-6 KO neonatal mice and characterized the immune responses. We found that IL-6 KO mice mounted significantly more anti-PPS14 IgG antibodies compared to wild-type neonates (Fig. 4a). In parallel to the increase in antibody responses, the frequencies of B220^+^GL7^+^Fas^+^ GC B cell (Fig. 4b and Supplementary Fig. 8a) and CD4^+^CXCR5^hi^PD-1^hi^FoxP3^−^ T_FH_ cell (Fig. 4c) populations were significantly higher in IL-6 KO mice than those in wild-type mice. Our IL-6 co-injection study had resulted with an increase in the T_FR_ population reciprocal to the decrease in T_FH_ cell frequency (Supplementary Fig. 7b)^14^. In support of a role for IL-6 in promoting T_FR_ cells, we measured significantly lower frequency of T_FR_ cells in immunized IL-6 KO mice compared to wild-type mice (Fig. 4d).

Next, we focused on IL-2 activity in the absence of IL-6 in immunized neonates. At seven dpi there was no difference in the frequency of CD4^+^IL-2^+^ population between the two mouse strains (Supplementary Fig. 8b). However, in IL-6 KO mice FoxP3^−^ T_FH_ cell population contained significantly less IL-2^+^ T_FH_ cells than the wild-type mice (Supplementary Fig. 8c). Like the ex vivo analyzed cells, in vitro stimulation of splenocytes from immunized neonatal mice with PMA/Ion also showed no difference in IL-2-producing CD4^+^ population between wild-type and IL-6 KO mice (Supplementary Fig. 8d) but there was a significant decrease in IL-2^+^ T_FH_ cell frequency in IL-6 KO mice after PMA/Ion stimulation (Fig. 4e). Importantly, in parallel to the decrease in IL-2 production, PMA/Ion stimulation resulted in higher frequency of T_FH_ population in IL-6 KO neonates compared to the T_FH_ population in wild-type neonates (Supplementary Fig. 8e).

The absence of systemic IL-6 also led to a change in the expression of IL-2 receptors on T_FH_ cells; both the receptors were downregulated in immunized IL-6 KO mice compared to wild-type mice (Fig. 4f, g). The decrease in the expression of IL-2 receptors was biologically meaningful because in vitro stimulation of splenocytes with IL-2 resulted in a significantly reduced frequency of p-STAT5^+^ T_FH_ cells in IL-6 KO mice than those from wild-type mice (Fig. 4h). Taken together, unlike in adult mice^29,32^, neonatal mice responses to vaccines improve in the absence of IL-6. Both the ablation of IL-2 mediated inhibition and the blunting of T_FR_ response likely contribute to the improved vaccine responses in neonatal IL-6 KO mice.

### CpG enhances vaccine response in neonatal mice through suppression of IL-6 and IL-2 signaling in TFH cells

The IL-6 co-injection and IL-6 KO mice studies highlighted the critical role for IL-6 in suppressing neonatal T_FH_ generation through the modulation of IL-2 activity. CpG improves vaccine responses in neonates by stimulating T_FH_ and GC B cell responses^13^. To test whether the improvement by CpG also involves the modulation of IL-6 and IL-2 activity, we characterized the immune responses in neonates immunized with CpG containing PPS14-TT or PPS14-TT vaccine in PBS. As shown previously^13^, CpG significantly increased serum IgG1 and IgG2c antibody levels against PPS14 (Supplementary Fig. 9a). CpG also increased the frequencies of splenic GC B cells (Supplementary Fig. 9b) and T_FH_ cells (Supplementary Fig. 9c) in neonates. Moreover, the increased frequency of T_FH_ cells was accompanied by decreased frequency of T_FR_ cells (Supplementary Fig. 9d) and T_FR_:T_FH_ ratio (Supplementary Fig. 9e, f).

**Fig. 5:**
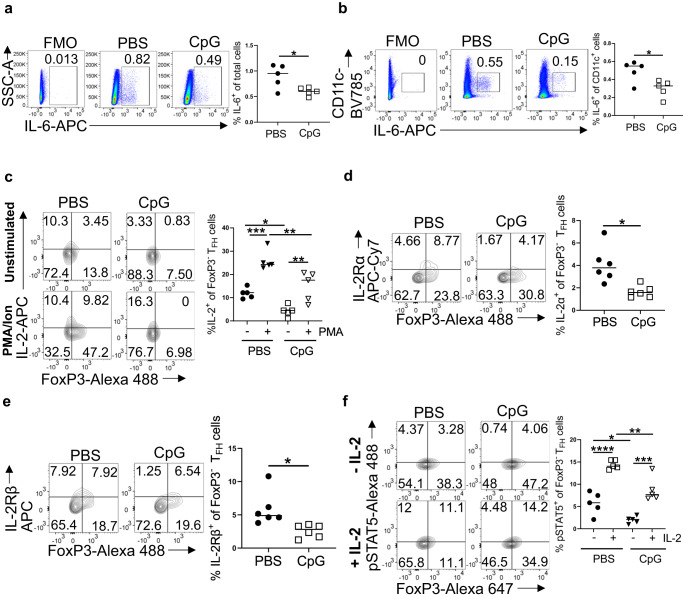
Neonatal mice germinal center response to CpG containing PPS14-TT vaccine. C57BL/6J mice were immunized i.p. with PPS14-TT (PBS) or PPS14-TT + CpG (CpG) and splenocytes were analyzed by FACS at 7 dpi. **a** Representative dot plots from 24 h post immunization depict the FMO control and the percentages of IL-6^+^ cells on total splenocytes. Mean percentages of IL-6^+^ cells among splenocytes are plotted (n = 5). **b** Representative dot plots from 24 h post immunization depict the FMO control and the percentages of IL-6^+^ cells on CD11c^+^ cells. Mean percentages of CD11c^+^IL-6^+^ subsets are plotted (*n* = 5). **c** Splenocytes from 7 dpi were in vitro stimulated with PMA/Ion for 4 h and T_FH_ cells were analyzed. Representative contour plots depict the percentages of IL-2^+^ cells among T_FH_ (CXCR5^hi^PD-1^hi^) population pre-gated on CD4^+^FoxP3^−^ cells. Mean percentages of IL-2^+^ cells among T_FH_ cells are plotted (*n* = 5). **d**, **e** Splenocytes from 7 dpi were pre-gated on CD4^+^CXCR5^hi^PD-1^hi^ T_FH_ cells. Representative contour plots depict percentages of IL-2Rα (**d**) or IL-2Rβ (**e**) expressing FoxP3^+^ and FoxP3^−^ cells on T_FH_ cells. Mean percentages of IL-2Rα^+^ and IL-2Rβ^+^ cells among T_FH_ cells are also plotted (*n* = 6). **f** Splenocytes from 7 dpi were stimulated with or without recombinant IL-2 for 15 min, followed by intracellular staining for p-STAT5. Representative contour plots depict the percentages of p-STAT5^+^ cells among FoxP3^+^ and FoxP3^−^ T_FH_ (pre-gated on CD4^+^CXCR5^hi^PD-1^hi^) cells. Mean percentages of p-STAT5^+^ cells among T_FH_ cells are plotted (*n* = 5). Experiments were performed twice. Unpaired student’s *t*-test and One-Way ANOVA were used for all comparisons; data represented as mean ± SEM are shown. *P* values < 0.05 were considered statistically significant. *P < 0.05, **P < 0.01, ***P < 0.001, ****P < 0.0001.

Next, we measured the production of IL-6 following immunization. Surprisingly, the percentages of IL-6^+^ splenocytes (Fig. 5a) as well as CD11c^+^ cells (Fig. 5b) were significantly lower in neonates immunized with CpG containing vaccine 24 h after vaccination. Ex vivo staining indicated that CpG containing PPS14-TT vaccine elicited significantly lower frequencies of IL-2-expressing CD4^+^ (Supplementary Fig. 10a) and FoxP3^−^ T_FH_ cells (Supplementary Fig. 10b) than those immunized with PPS14-TT alone. As with ex vivo stained cells, there were lower frequencies of IL-2-expressing CD4^+^ (Supplementary Fig. 10c) and T_FH_ cells (Fig. 5c) following PMA/Ion stimulation of splenocytes from neonates immunized with CpG containing PPS14-TT vaccine compared to those immunized with PPS14-TT alone. More importantly, the PMA/Ion induced increase in IL-2 production correlated with the lower frequency of T_FH_ cells (Supplementary Fig. 10d). There was also a correlation between the reduced production of IL-2 in neonates immunized with the CpG containing vaccine and higher frequency of T_FH_ cells compared to neonates immunized with PPS14-TT alone.

Inclusion of CpG in PPS14-TT vaccine also influenced the expression of IL-2 receptors. Both, IL-2Rα^+^ and IL-2Rβ^+^ T_FH_ cell frequencies were significantly lowered by CpG (Fig. 5d, e). The decrease in the expression of IL-2 receptors had functional consequence because stimulation of splenocytes from CpG containing vaccine led to lower frequencies of p-STAT5^+^ T_FH_ cells than those immunized with PPS14-TT alone following the stimulation of purified splenocytes with IL-2 (Fig. 5f). Thus, CpG improves vaccine responses by protecting T_FH_ cells from the inhibitory activity of IL-2 through the reduction of IL-6 and IL-2 production and by decreasing IL-2Rα and IL-2Rβ expression on T_FH_ cells.

**Fig. 6:**
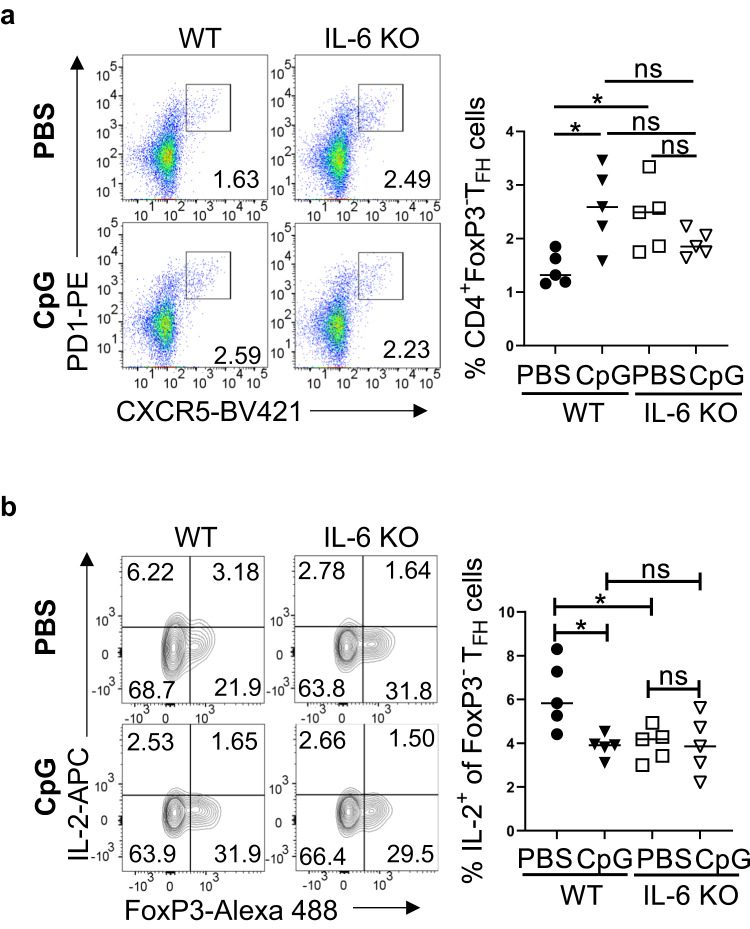
IL-6 KO neonatal mice T_FH_ response to CpG containing PPS14-TT vaccine immunization. Neonatal wild-type (C57BL/6J) and IL-6 KO mice were immunized i.p. with PPS14-TT (PBS) or PPS14-TT + CpG (CpG) and splenocytes were analyzed by FACS at 7 dpi. **a** Representative dot plots depict the percentages of T_FH_ (CXCR5^hi^PD-1^hi^) cells pre-gated on CD4^+^FoxP3^−^ cells. Mean percentages of T_FH_ cells are plotted (*n* = 5). **b** Representative contour plots depict the percentages of IL-2^+^ cells among T_FH_ (CXCR5^hi^PD-1^hi^) population pre-gated on CD4^+^FoxP3^−^ cells. Mean percentages of IL-2^+^ cells among T_FH_ cells are plotted (*n* = 5). Unpaired student’s *t*-test and One-Way ANOVA were used for all comparisons; data represented as mean ± SEM are shown. *P* values < 0.05 were considered statistically significant. *P < 0.05 and ns (non-significant).

Finally, to assess the relative contribution of the decrease in IL-6 production by the splenic cells in the improvement of neonatal T_FH_ cells immunized with CpG containing PPS14-TT vaccine, we immunized IL-6 KO neonates with the CpG containing vaccine and compared the T_FH_ response to IL-6 KO neonates immunized with PPS14-TT vaccine alone and the wild-type neonates immunized with the CpG containing vaccine. We found that, unlike in wild-type mice, inclusion of CpG to PPS14-TT vaccine did not improve the T_FH_ response in IL-6 KO mice over those immunized with PPS14-TT alone (Fig. 6a). The levels of T_FH_ cells in wild-type mice immunized the CpG containing vaccine and the IL-6 KO mice immunized with and without the CpG containing vaccine were comparable. Moreover, the frequency of T_FH_ cells in these three groups of mice were significantly higher than the wild-type mice immunized with PPS14-TT alone. Confirming the previously observed association between the levels of splenic IL-6 producing cells and the IL-2^+^ T_FH_ cells, we measured significantly lower levels of IL-2^+^ T_FH_ cells in all three groups and the decrease in IL-2^+^ T_FH_ cells were comparable between the three groups of neonates (Fig. 6b). Thus, when IL-6 is absent, the CpG containing vaccine cannot improve T_FH_ response more than the improvement in wild-type neonatal mice.

## Discussion

In adults, the formation of CXCR5^hi^PD-1^hi^ FoxP3^−^ CD4 T helper cells from lymphoid organs is essential for the generation GC B cells and antibodies against vaccine antigens^15,18^. We and others have shown that the frequency of the same population is substantially smaller in immunized neonates^3,13,14,27^. Throughout this study, the lowest number of flow cytometric events measured for CD4^+^CXCR5^hi^PD-1^hi^ FoxP3^−^ T_FH_ cells in immunized neonates was 98 and the average number was 240. For adults, the lowest number of events for the same population was 205 and the average number was 698. Thus, the inability of neonates to develop fully mature GC reaction is likely responsible for their suboptimal vaccine responses. In search of the mechanism(s) responsible for the suboptimal T_FH_ response in neonates, we previously reported suppression of neonatal T_FH_ and antibody responses when IL-6 is co-injected with PPS14-TT vaccine^14^. This is in sharp contrast to adult mice, which mount increased antibody^14,30^ and T_FH_^14^ responses with excess IL-6. Here, we showed that excess IL-6 sensitizes T_FH_ cells to the inhibitory cytokine IL-2 by increasing the expression of IL-2 receptors on T_FH_ cells and by inducing the production of IL-2 by T_FH_ cells. Conversely, vaccine response is improved in neonatal IL-6 KO mice. The improvement in IL-6 KO mice is accompanied by dampened IL-2 production and reduced IL-2 receptor expression on T_FH_ cells. Underscoring this unique mechanism, we showed that CpG containing vaccine improved T_FH_ response while simultaneously decreasing IL-6 and IL-2 production and suppressing IL-2Rα and IL-2Rβ expression on T_FH_ cells.

The inhibitory activity of IL-2 on T_FH_ cells is well established^15,24^. In the current study, we focused on the regulation of IL-2 mediated suppression of neonatal T_FH_ cells because in adult mice the expansion of T_FH_ cells is dependent on IL-6 mediated downregulation of IL-2Rβ on T_FH_ cells which limits STAT5 phosphorylation^29^. We first observed that immunized neonatal mice spleens contained significantly more IL-6 producing splenocytes as well as CD11c^+^, F4/80^+^, CD19^+^ and CD3^+^ cells than the adult mice. Although IL-6 producing cell frequency peaked in 24 h and decreased thereafter in both the age groups, the increases in neonatal cells were substantially more than those of adult cells. In a recent study, Pyle et al. reported increased IL-2 production in neonatal pulmonary lymph nodes following respiratory syncytial virus infection^28^. Highlighting the role for IL-2 in the suppression of T_FH_ cells, they found that inhibition of IL-2 led to expanded T_FH_ population in infected neonatal mice, as was shown previously in adult mice^38,40^. These authors also showed higher expression of IL-2Rα and IL-2Rβ on naive CD44^-^CD62L^+^FoxP3^−^CD4^+^ cells and elevated p-STAT5 activity in CD62L^+^FoxP3^−^CD4^+^ cells following in vitro stimulation with IL-2. However, they did not assess the regulation of IL-2 receptor expression and p-STAT5 activation in infected or immunized neonatal mice T_FH_ cells and they concluded that the increased receptor expression in naive neonatal mice CD4^+^ cells is unlikely to play a role in heightened IL-2 activity in neonatal CD4^+^ cells. Like reported by Pyle et al.^28^, we also measured higher expression of IL-2 by naive in addition to immunized neonatal mice CD4^+^ cells. In a recent study, Papillion and colleagues showed that IL-2 produced by T_FH_ cells is especially instrumental for the inhibition of T_FH_ cells^29^. Further gating of CD4^+^ cells from immunized neonates for FoxP3^−^CXCR5^hi^PD-1^hi^ T_FH_ cells indicated that IL-2 production was especially elevated in this population. Moreover, we measured higher frequencies of IL-2Rα and IL-2Rβ expressing T_FH_ cells in neonatal mice compared to adult mice following immunization. The increase in IL-2 receptor positive T_FH_ cells in immunized neonates was biologically relevant because, compared to adult cells, in vitro stimulation of neonatal CD4^+^ cells resulted in higher frequency of p-STAT5^+^ T_FH_ cells, which is associated with negative regulation of T_FH_ response^38,40^.

Since IL-6 was expressed higher in immunized neonates along with heightened IL-2 signaling in T_FH_ cells, we investigated the link between IL-6 and IL-2 activity on neonatal T_FH_ cells. To assess the regulation of IL-2 activity when IL-6 is absent and when IL-6 is in excess, we immunized IL-6 KO mice in addition to repeating the IL-6 co-injection study^14^. The results of these two complementary experiments verified the association between IL-6 and IL-2 production as well as IL-2 receptor expression on T_FH_ cells. In neonatal mice, IL-2-expressing T_FH_ cell frequency was substantially increased when IL-6 was co-injected and decreased when IL-6 was absent. This inverse relationship was also present for the expression of IL-2 receptors and STAT5 phosphorylation as well as in T_FH_ generation. Corroborating the IL-6 co-injection and IL-6 KO mice immunization studies, CpG mediated improvement in T_FH_, GC B cell and antibody responses was accompanied by diminished IL-6 and IL-2 production, together with reduced IL-2Rα and IL-2Rβ expression as well as blunted STAT5 phosphorylation. The decrease in IL-6^+^ splenic cell population in neonates immunized with the CpG containing vaccine is intriguing since CpG is a well-known inducer of inflammatory cytokines, including IL-6, when cells are stimulated in vitro^41^. A possible explanation for this surprising outcome is the likely differences in the in vivo environment between neonatal and adult mice as was shown by Mastelic and colleagues who reported that adoptively transferred adult CD4^+^ cells did not expand in neonatal mice following immunization^13^. Conversely, they showed that the transfer of neonatal CD4^+^ cells into adult recipients led to the expansion of donor CD4^+^ cells in adult mice. Thus, neonatal environmental factors may be regulating the induction of IL-6 production by CpG when given with the PPS14-TT vaccine.

The importance of the decrease in IL-6^+^ splenic cells in the improvement of GC response following the immunization of wild-type neonates with CpG containing PPS14-TT vaccine is further underscored in our experiments where IL-6 KO neonates were immunized with the CpG containing vaccine. The fact that immunization of IL-6 KO with the CpG containing vaccine did not further improve the T_FH_ population beyond IL-6 KO neonates immunized with PPS14-TT alone or the wild-type neonates immunized with the CpG containing vaccine suggests that the downregulation of IL-6 by CpG in wild-type neonates is the main mechanism responsible for the suppression of IL-2 and the improvement of T_FH_ cells.

At this point, it is not clear how IL-6 elicits a divergent function in regulating IL-2 production and IL-2 receptor expression on T_FH_ cells of adult and neonatal mice. Nevertheless, the unveiling of IL-6-mediated suppression of neonatal vaccine responses that involve enhanced IL-2 activity on T_FH_ cells has implications for the development of vaccines targeting early age. Adjuvants that improve vaccine responses in adults through enhanced production of IL-6 may not be suitable for neonates and infants. For example, SARS-CoV-2 mRNA vaccines contain lipid nanoparticles (LNP), which are shown to improve host immune response through the production of IL-6 and the expansion of T_FH_ in adult mice^32,33^. To our knowledge, efficacy of these vaccines in infants younger than 6 months of age have not been reported. If the mRNA-LNP vaccines stimulate IL-6 production in neonates also, these vaccines may not elicit protective antibodies in this age group due to enhanced sensitization of T_FH_ cells to IL-2. Taken together, our findings support the tailoring of vaccines intended for early age based on the unique properties of this age group.

## Methods

### Mice

Wild-type and IL-6 KO (B6.129S2-Il6(tm1Kopf/J)) mice with a C57BL/6 genetic background were purchased from Jackson Laboratory (Bar Harbor, Maine), bred, and kept in pathogen-free animal facilities in accordance with FDA Center for Veterinary Medicine guidelines. Neonatal (5- to 7-day-old) and adult (6- to 10-week-old) mice were used for immunization experiments. All animal procedures were approved by FDA’s Institutional Animal Care and Use Committee (Protocol 2017-48). For euthanasia, adult and neonatal mice were exposed to CO_2_ inhalation. CO_2_ was introduced at a rate of at least 30% chamber volume per minute. CO_2_ inhalation was followed by cervical dislocation for adult mice and decapitation for neonatal mice. Anesthesia was induced by administering isoflurane, set at 3–4% for 1–2 min in the induction box and the flow rate of O_2_ is set at 1.0 L/min.

### Immunization

Tetanus toxoid conjugated type 14 pneumococcal polysaccharide (PPS14-TT) vaccine was manufactured as described^42^. PPS14-TT vaccine was emulsified with aluminum hydroxide [Al(OH)_3_] (Thermo Fischer, Waltham, MA). Aluminum hydroxide constituted 1/4th of injection volume. For IL-6 co-injection experiments, PPS14-TT together with recombinant IL-6 (500 ng/adult and 100 ng/neonate (R&D systems, Minneapolis, MN)) was emulsified with aluminum hydroxide. The adjuvant CpG 1826 (TCCATGA*CG*TTCCTGACGTT) was synthesized at FDA core facility. The CpG (10 μg per neonate mouse) containing PPS14-TT vaccine was emulsified with aluminum hydroxide by stirring for 30 min prior to injection. One and 0.5 μg of vaccines were injected in 150 μl and 30 μl volumes i.p. per adult and neonatal mice, respectively.

### Antibody for FACS analysis

Single-cell suspensions were prepared from splenocytes. Dead cells were stained by incubating cell suspensions with Zombie Aqua (BioLegend, Cat # 423102) diluted at 1:1000 dilution in PBS for 15 min at room temperature. Cells were washed and stained using FACS buffer containing 2% FBS, 0.5 M EDTA in PBS. The following antibodies were used for surface staining at room temperature for 30 min: α-CD4 (BioLegend, 1:100 dilution, clone GK1.55, Cat # 100434), α-B220 (BioLegend, 1:100 dilution, clone RA3-6B2, Cat # 103244), α-PD-1 (BD Biosciences, 1:100 dilution, clone J43 Cat # 566831 or BioLegend, 1:100 dilution, clone 29F.1A12, Cat # 135206), α-CXCR5 biotin (BD Biosciences, 1:100 dilution, clone 2G8, Cat # 551960), α-GL7 (BioLegend, 1:100 dilution, clone GL-7, Cat # 144612), α-CD95 (BD Biosciences, 1:100 dilution, clone J02, Cat # 563647), α-IL-2Rα (BioLegend, 1:100 dilution, clone PC61, Cat # 102026), α-IL-2Rβ (BioLegend, 1:100 dilution, clone TM-β1, 123214), α-CD19 (BioLegend, 1:100 dilution, clone 6D5, Cat # 115523), α-CD3 (BioLegend, 1:100 dilution, clone 17A2, Cat # 100204), α-CD11c (BioLegend, 1:100 dilution, clone N418, Cat # 117336). To detect biotinylated CXCR5, cells were further incubated with streptavidin-BV-421 (BD Biosciences, 1:100 dilution, Cat # 563259) for 30 min at room temperature. For intracellular staining, samples were fixed with the FoxP3 Fix/Perm buffer set, following the manufacturer’s (ThermoFisher Scientific, eBioscience, Waltham, MA) instructions. Samples were then intracellularly stained with α-FoxP3 (BD Biosciences, 1:100 dilution, clone MF23, Cat # 560401 or BioLegend, 1:100 dilution, clone 150D, Cat # 320012), α-IL-2 (BD Biosciences, 1:100 dilution, clone JES6-5H4, Cat # 554429) or α-IL-6 (BioLegend, 1:100 dilution, clone MP5-20F3, Cat # 504508) antibodies for 30 min at room temperature. Flow cytometry data were acquired on Fortessa or Fortessa X20 flow cytometers (BD Biosciences) and analyzed using the FlowJo software v10.8.1 (FlowJo, Ashland, OR).

### In vitro stimulation of cells

For the assessment of IL-2 production, single-cell suspensions of splenocytes were stimulated with or without PMA (25 ng/ml, Sigma-Aldrich) and ionomycin (500 ng/ml, Invitrogen) in the presence of Brefeldin A (1:1000, Invitrogen) at 37 °C for 4 h. After incubation, dead cells were stained with Zombie Aqua for 15 min at room temperature followed by surface marker staining. The cells were then fixed and permeabilized with FoxP3 Fix/Perm buffer set (ThermoFisher) and incubated with antibodies for α-IL-2 (BD Biosciences, 1:100, JES6-5H4) for 30 min at room temperature and analyzed in flow cytometry.

### Phospho proteins (p-STAT5 and p-STAT3) FACS Analysis

For p-STAT5 measurement, single-cell suspensions were incubated in culture media supplemented with 10% FBS, alone or with mouse recombinant IL-2 (R&D Systems, 50 ng/ml) for 15 min at 37 °C. After washing with FACS buffer containing 1% FBS with 1 mM EDTA, cells were first fixed with BD Cytofix fixation buffer (BD Biosciences) for 10 min at 37 °C, and then permeabilized with pre-chilled BD Phosphoflow buffer III (BD Biosciences) for 10 min at 4 °C. Cell surface antibodies and p-STAT5 (BD Biosciences, 1:100 dilution, pY694, clone 47, Cat # 612598) antibody were incubated together in FACS buffer for 30 min at room temperature. To detect biotinylated CXCR5, cells were further incubated with streptavidin-BV421 (BD Biosciences, 1:100 dilution) for 30 min at room temperature. For detection of T_FR_ cells, cells were washed and FoxP3 antibody was added to the permeabilization buffer (eBioscience) for 30 min at room temperature. For p-STAT3 staining, the cells were fixed and permeabilized as described for p-STAT5. Cell surface antibodies and p-STAT3 (BioLegend, 1:100 dilution, pY705, clone 13A3-1, Cat # 651006) antibody were incubated together in FACS buffer for 30 min at room temperature followed by CXCR5 and FoxP3 staining.

### Measurement of antibody titers against PPS14

Serum antibody levels were measured in ELISA 4 weeks post immunization. For antibody measurement, 96-well plates were coated with purified PPS14 (ATCC, Manassas, Virginia) at 10 μg/ml in PBS (pH of 7) for 2 h at room temperature and then blocked for 1 h at room temperature with 5 % neonatal calf serum (Millipore Sigma, St. Louis, MO) in PBS. Serum samples (1:20 dilution) were serially diluted and 100 μl of diluted samples were transferred on coated plates for overnight incubation. After washing, wells were incubated with horseradish peroxidase-conjugated goat anti-mouse IgG-Fc or IgA antibody (Bethyl Laboratories, Waltham, MA) for 3 h at room temperature. For detection, 100 μl of KPL SureBlue TMB microwell peroxidase substrate (Seracare, Gaithersburg, MD) is added to the wells and incubated for 15–30 min followed by addition of stop solution (KPL TMB BlueSTOP solution, Seracare). The absorbance is measured at 450 nm.

### Statistical analysis

All statistical analyses were performed using Prism 9 (GraphPad). Unpaired student’s *t*-test and One-way ANOVA was used for all comparisons; data represented as mean ± SEM are shown. *P* values < 0.05 were considered statistically significant. *P < 0.05, **P < 0.01, ***P < 0.001, ****P < 0.0001. The data points in the graphs represent measurements taken from distinct samples in one experiment.

### Reporting summary

Further information on research design is available in the Nature Research Reporting Summary linked to this article.

### Supplementary information

Supplemental Figures

Reporting Summary

## Data Availability

All data generated or analyzed during this study are included in this published article (and its supplementary information files).
