# Supplementary material for: IL6 suppresses vaccine responses in neonates by enhancing IL2 activity on T follicular helper cells
Source: NPJ Vaccines. 2023 Nov 8;8:173. doi: 10.1038/s41541-023-00764-1 (PMC10632457; doi:10.1038/s41541-023-00764-1)
Supplement: Supplementary file 1 — Supplemental Figures [file 41541_2023_764_MOESM1_ESM.pdf]

Supplemental Figures

Supp. Fig. 1

a **Gating strategy for T_{FH} cells**

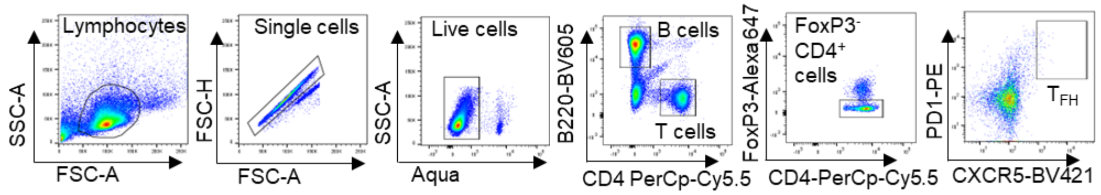

b

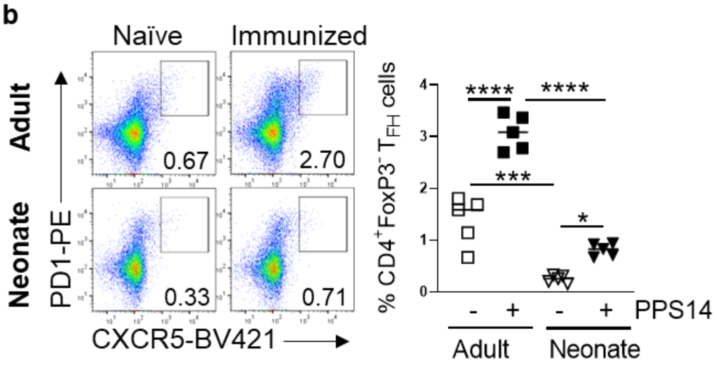

c **Gating strategy for IL-6 on different cell subsets**

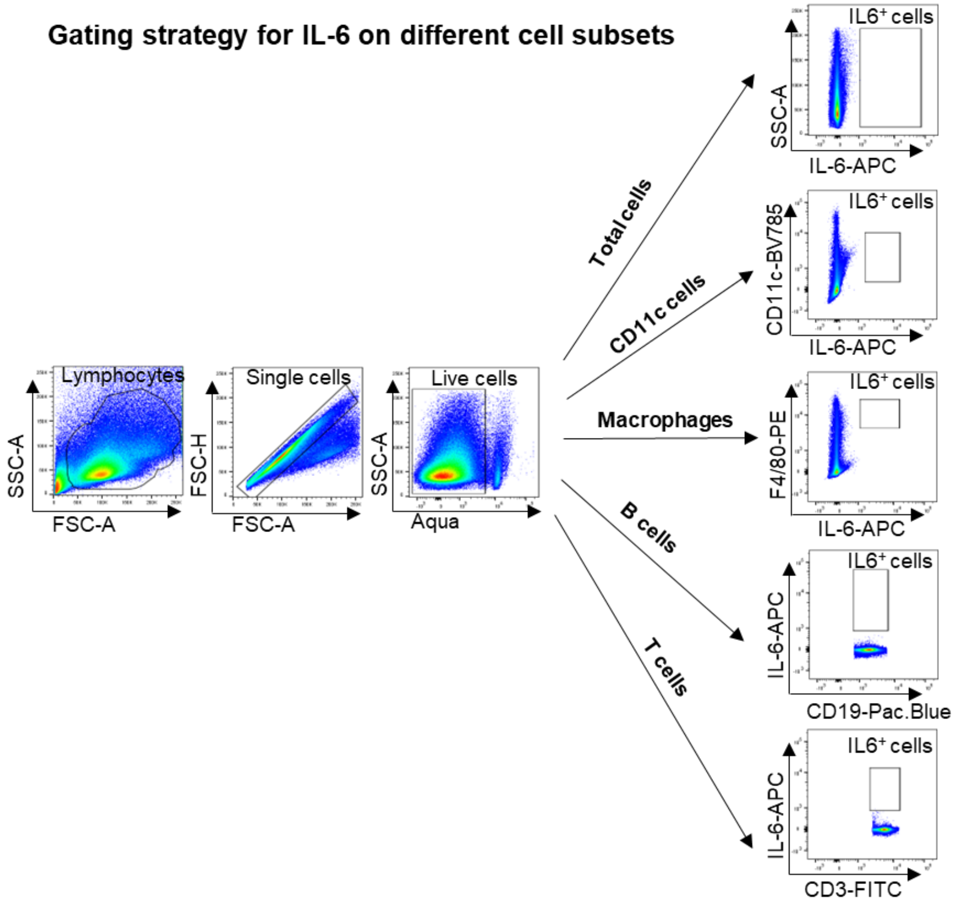

Supplemental Figure 1. (a and b) detection of T_{FH} cells in immunized neonatal and adult mice. Adult (6- to 10-week-old) and neonatal (5- to 7-day-old) mice were immunized i.p. with PPS14-TT and splenocytes were analyzed by FACS. **(a)** Gating strategy for the detection of T_{FH} cells in splenocyte pools by FACS. Dead cells were excluded with LIVE/DEAD (Aqua) dye and cells were further gated with antibodies against CD4, B220, PD-1, CXCR5 and FoxP3. T_{FH} cells were detected within the $CD4^+$ T cells as $FoxP3^-CXCR5^{hi}PD\text{-}1^{hi}$ population. **(b)** Representative dot plots from naïve and immunized mice depict the percentages of T_{FH} ($CXCR5^{hi}PD\text{-}1^{hi}$) cells pre-gated on $CD4^+FoxP3^-$ cells 7 dpi. Mean percentages of T_{FH} cells are plotted (n=5). **(c)** Gating strategy for the detection of $IL\text{-}6^+$ cells in splenocyte pools by FACS. Dead cells were excluded with LIVE/DEAD (Aqua) dye and IL-6 producing cells were identified by using antibodies against CD11c, F4/80, CD19, and CD3. Experiments were performed twice. One-Way ANOVA was used for all comparisons; data represented as mean +/- SEM are shown. P values <0.05 were considered statistically significant. *P<0.05, ***P<0.001, ****P<0.0001.

Supp. Fig. 2

a **CD11c cells**

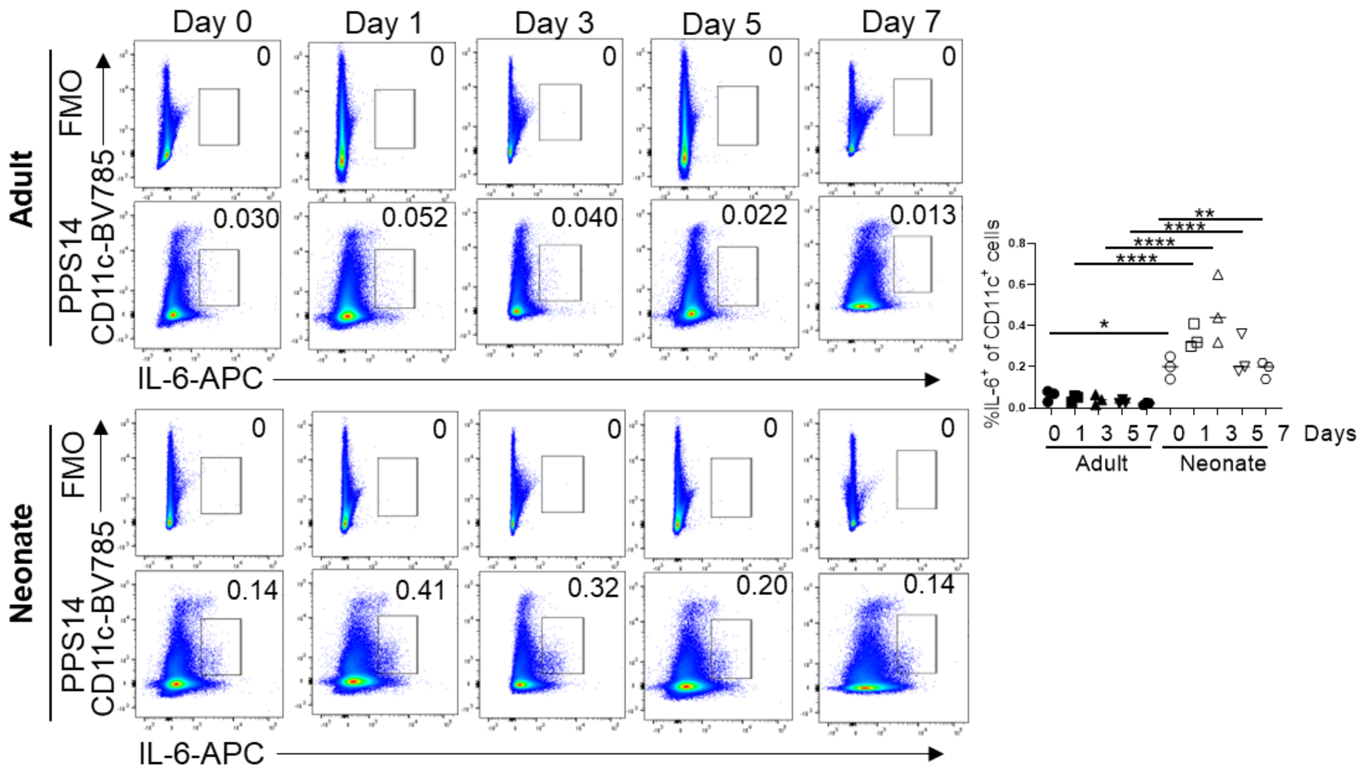

b **Macrophages**

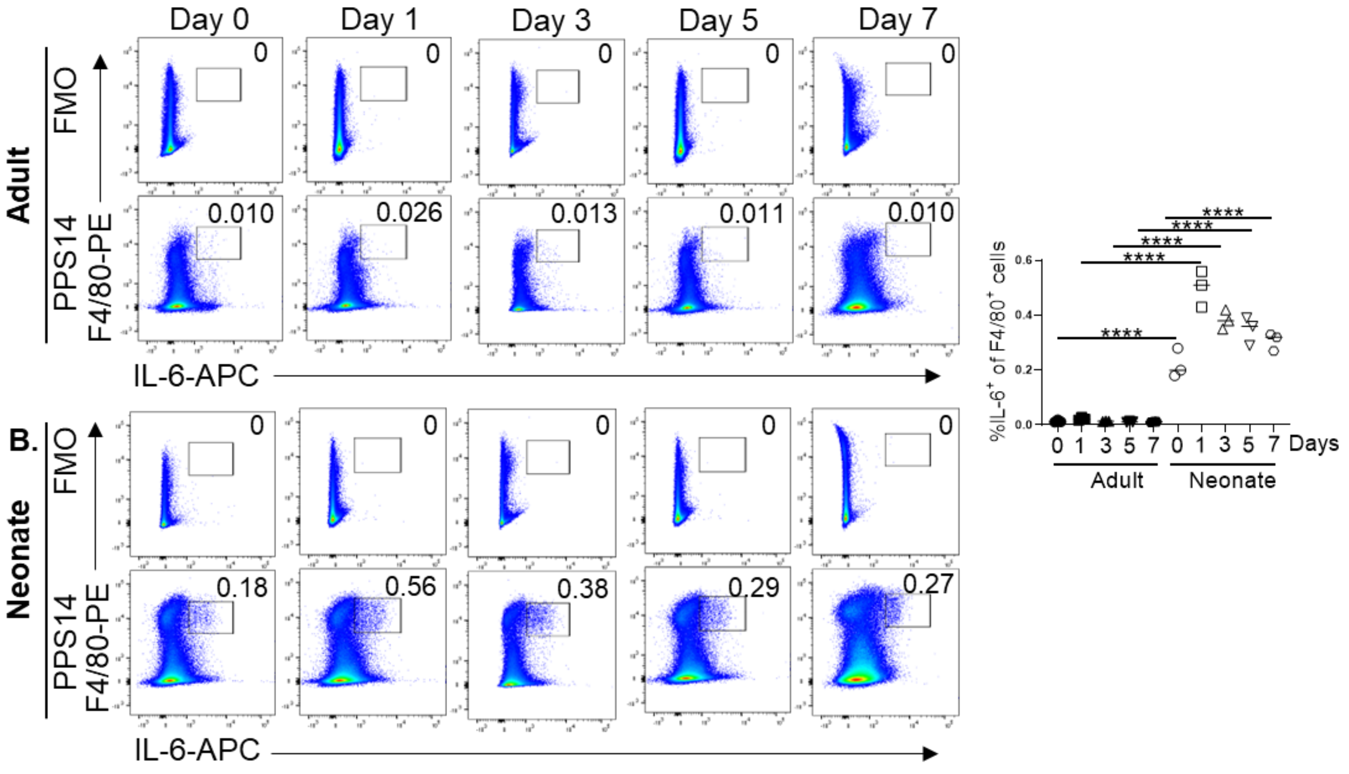

Supplemental Figure 2. (a). Adult (6- to 10- week-old) and neonatal (5- to 7- day-old) mice were immunized i.p. with PPS14-TT and splenocytes were analyzed by FACS. Splenic cells were analyzed 0, 1, 3, 5, and 7 dpi. Representative dot plots depict the Fluorescence minus one (FMO) control and the percentages of $IL\text{-}6^{+}CD11c^{+}$ cells. FMOs for each age group are the same for Day 1 and Day 5 because the samples for these days were analyzed on the same day. Also, FMOs for adult and neonates are the same for Day 3 because cells from adult and neonatal mice were pooled due to insufficient number of cells for each age group on this time point. Mean percentages of $IL\text{-}6^{+}$ cells among $CD11c^{+}$ cells are plotted (n=3). **(b)** Representative dot plots depict the FMO control and the percentages of $IL\text{-}6^{+}$ cells gated on $F4/80^{+}$ cells. FMOs for each age group are the same for Day 1 and Day 5 because the samples for these days were analyzed on the same day. Also, FMOs for adult and neonates are the same for Day 3 because cells from adult and neonatal mice were pooled due to insufficient number of cells for each age group on this time point. Mean percentages of $IL\text{-}6^{+}$ cells on $F4/80^{+}$ cells are plotted (n=3). Experiments were performed two times. One-Way ANOVA was used for all comparisons; data represented as mean +/- SEM are shown. P values <0.05 were considered statistically significant. *P<0.05, **P<0.01, ****P<0.0001.

Supp. Fig. 3

a **B cells**

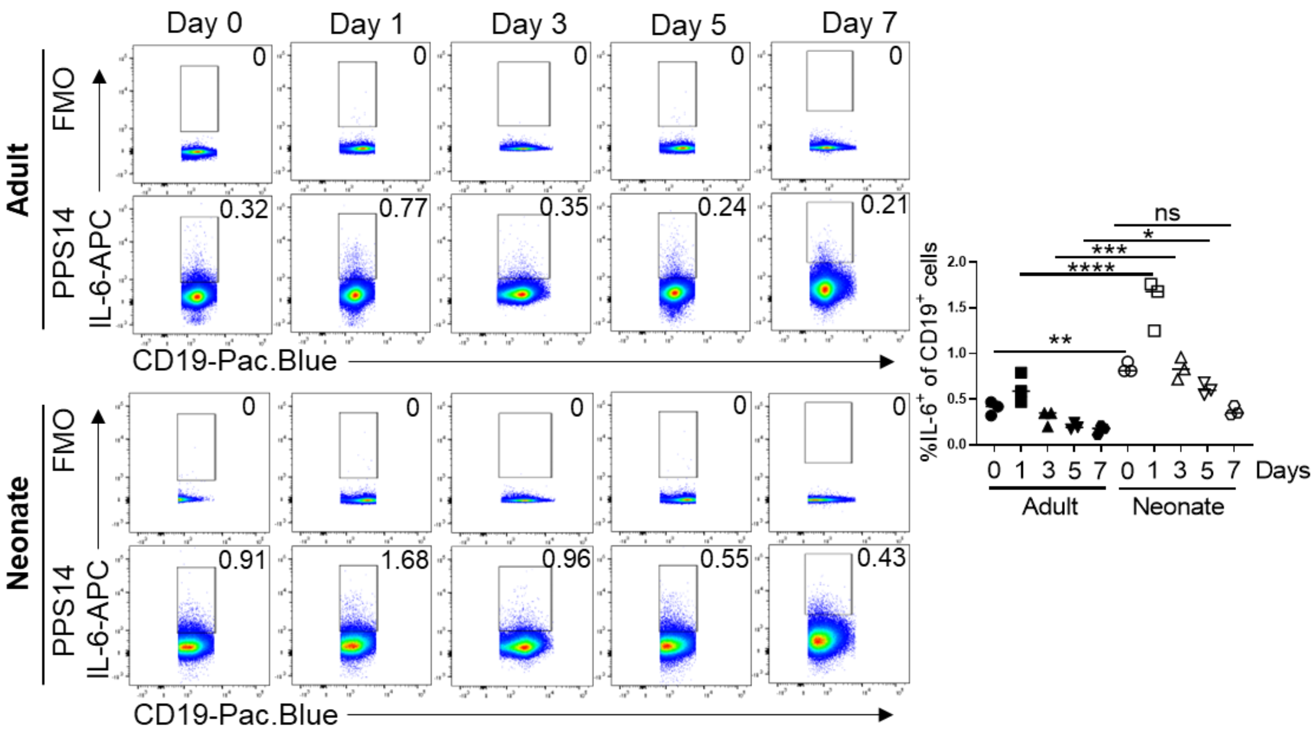

b **T cells**

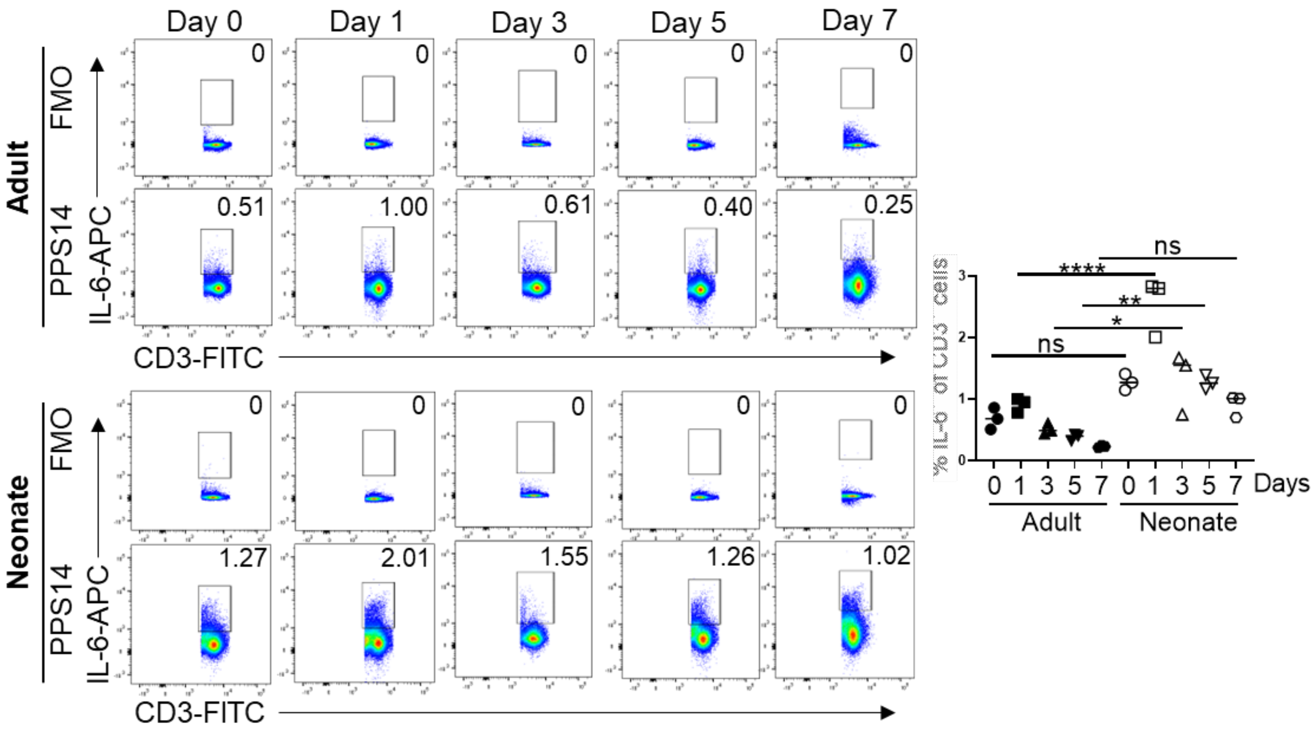

Supplemental Figure 3. (a) Adult (6- to 10- week-old) and neonatal (5- to 7- day-old) mice were immunized i.p. with PPS14-TT and splenocytes were analyzed by FACS. Splenic cells were analyzed 0, 1, 3, 5, and 7 dpi. Representative dot plots depict the depict the FMO control and the percentages of IL-6^{+} cells gated on CD19^{+} cells. FMOs for each age group are the same for Day 1 and Day 5 because the samples for these days were analyzed on the same day. Also, FMOs for adult and neonates are the same for Day 3 because cells from adult and neonatal mice were pooled due to insufficient number of cells for each age group on this time point. Mean percentages of IL-6^{+} cells on CD19^{+} cells are plotted (n=3). **(b)** Representative dot plots depict the depict the FMO control and the percentages of IL-6^{+} cells gated on CD3^{+} cells. FMOs for each age group are the same for Day 1 and Day 5 because the samples for these days were analyzed on the same day. Also, FMOs for adult and neonates are the same for Day 3 because cells from adult and neonatal mice were pooled due to insufficient number of cells for each age group on this time point. Mean percentages of IL-6^{+} cells on CD3^{+} cells are plotted (n=3). Experiments were performed two times. One-Way ANOVA was used for all comparisons; data represented as mean +/- SEM are shown. P values <0.05 were considered statistically significant. *P<0.05, **P<0.01, ***P<0.001, ****P<0.0001 and ns (non-significant).

Supp. Fig. 4

a **Gating strategy for pSTAT3$^+$ on CD4$^+$ cells**

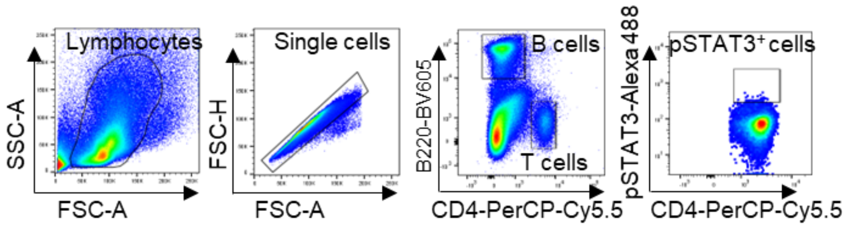

b **Gating strategy for pSTAT3$^+$ on Total T_{FH} cells (PD1$^+$CXCR5$^+$) and Pre-T_{FH} (PD1^{int}CXCR5^{int}) cells**

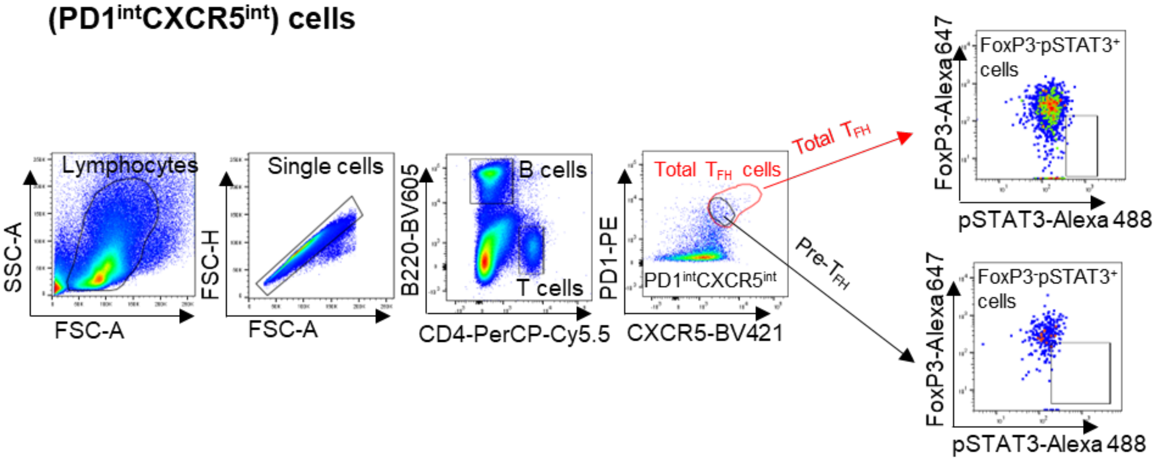

c **Gating strategy for FoxP3$^-$pSTAT3$^+$ on CD4$^+$ cells**

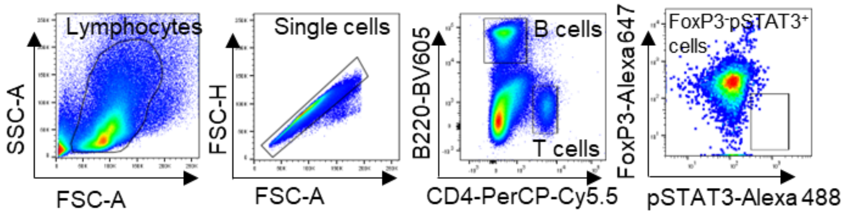

d

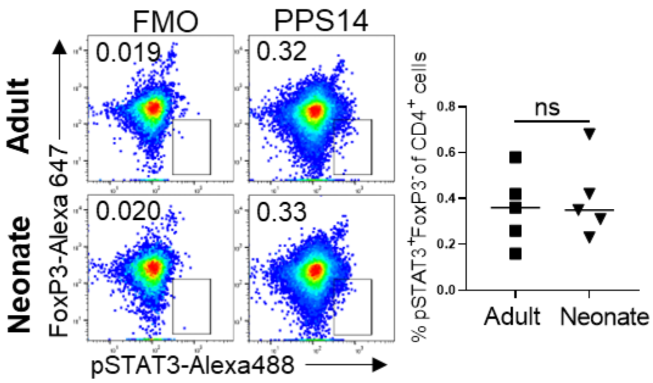

Supplemental Figure 4. (a) Gating strategy for the detection of p-STAT3$^+$ cells on T cells in splenocyte pools by FACS. The cells were stained with p-STAT3, B220 and CD4 antibodies and p-STAT3$^+$ T cells were detected within the singlets as p-STAT3$^+$CD4$^+$ population. **(b)** Gating strategy for the detection of p-STAT3$^+$ cells on total T_{FH} and pre-T_{FH} cells in splenocyte pools by FACS. The cells were stained with CD4, B220, PD-1, CXCR5, FoxP3 and p-STAT3 antibodies and p-STAT3$^+$ total T_{FH} cells were detected within the CD4$^+$ cells as PD-1$^+$CXCR5$^+$FoxP3$^-$p-STAT3$^+$ population. Also, p-STAT3$^+$ pre-T_{FH} cells were detected within the CD4$^+$ cells as PD-1^{int}CXCR5^{int}FoxP3$^-$p-STAT3$^+$ population. **(c)** Gating strategy for the detection of FoxP3$^-$p-STAT3$^+$ cells on T cells in splenocyte pools via flow cytometry. The cells were stained with CD4, B220, FoxP3, and p-STAT3 antibodies and Foxp3$^-$p-STAT3$^+$ cells were detected within the singlets as Foxp3$^-$p-STAT3$^+$CD4$^+$ population. **(d)** Adult (6- to 10- week-old) and neonatal (5- to 7- day-old) mice were immunized i.p. with PPS14-TT and 24 hours pi splenocytes were analyzed for p-STAT3 by FACS. Splenocytes were pre-gated on CD4$^+$ cells and the percentage of FoxP3$^-$p-STAT3$^+$ cells were analyzed. Representative FACS plots depict the percentages of FoxP3$^-$p-STAT3$^+$ cells on CD4$^+$ cells. Mean percentages of FoxP3$^-$p-STAT3$^+$ cells among CD4$^+$ cells are plotted (n=5). Experiments were performed three times. Unpaired student's t-test was used for all comparisons; data represented as mean +/- SEM are shown. P values <0.05 were considered statistically significant and ns (non-significant).

Supp. Fig. 5

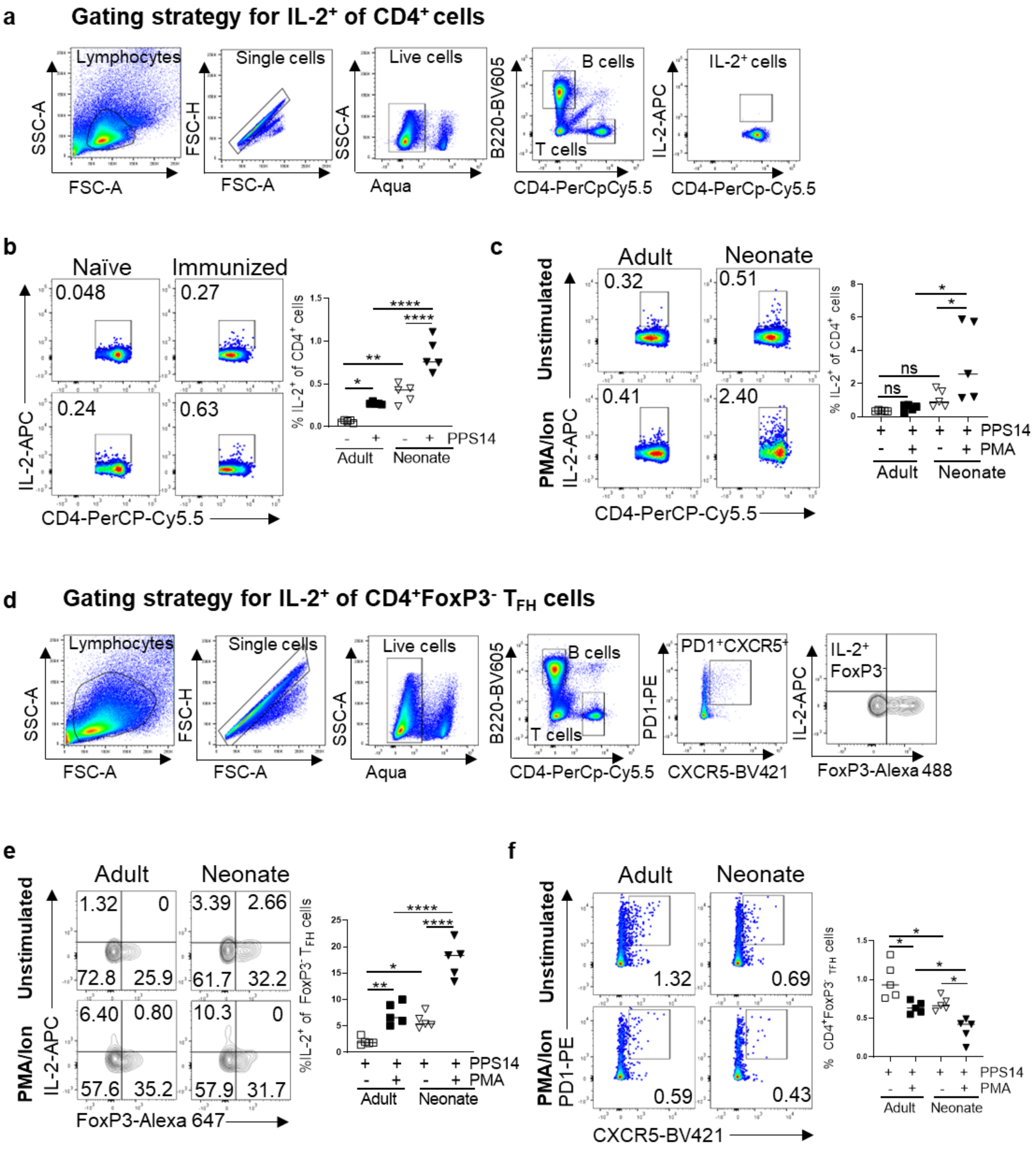

Supplemental Figure 5. (a and b) Analysis of IL-2-expressing T cells in immunized mice by FACS. Adult (6- to 10-week-old) and neonatal (5- to 7-day-old) mice were

immunized i.p. with PPS14-TT and splenocytes were analyzed by FACS. **(a)** Gating strategy for the detection of IL-2^+ cells splenic CD4+ T cells. Dead cells were excluded with LIVE/DEAD (Aqua) dye and cells were further stained with antibodies against CD4, B220, and IL-2. IL-2^-expressing T cells were detected as IL-2^+CD4^+ population. **(b)** Representative dot plots depict the percentages of IL-2-expressing CD4^+ cells from naïve and PPS14-TT immunized mice. Mean percentages of IL-2^+ among CD4^+ cells are plotted (n=5). **(c, e and f)** Splenocytes from immunized adult and neonatal mice were in vitro stimulated with PMA/Ion for 4 hours and cells were analyzed by FACS. **(c)** Representative dot plots depict the percentages of IL-2-expressing CD4^+ cells from unstimulated and PMA/Ion splenocytes. Mean percentages of IL-2^+ among CD4^+ cells are plotted (n=5). **(d)** Gating strategy for the detection of IL-2^+-expressing splenic T_{FH} cells by FACS. Dead cells were excluded with LIVE/DEAD (Aqua) dye and cells were further stained with antibodies against CD4, B220, PD-1, CXCR5, FoxP3, and IL-2 antibodies. IL-2-expressing T_{FH} cells were detected within the CD4^+ cells as CXCR5^{hi}PD-1^{hi}Foxp3^-IL-2^+ population respectively. **(e)** Representative counter plots depict the percentages of IL-2-expressing FoxP3^+ and FoxP3^- cells pre-gated on T_{FH} (CD4^+CXCR5^{hi}PD-1^{hi}) population. Mean percentages of IL-2^+ cells among FoxP3^- T_{FH} cells are plotted (n=5). **(f)** Representative dot plots depict the percentage of T_{FH} (CXCR5^{hi}PD-1^{hi}) cells pre-gated on CD4^+FoxP3^- cells. Mean percentages of T_{FH} cells are plotted (n=5). Experiments were performed twice. One-Way ANOVA was used for all comparisons; data represented as mean +/- SEM are shown. P values <0.05 were considered statistically significant. *P<0.05, **P<0.01, ****P<0.0001 and ns (non-significant).

Supp. Fig. 6

a **Gating strategy for IL-2Rα⁺ or IL-2Rβ⁺ of CD4⁺FoxP3⁻ T_{FH} cells**

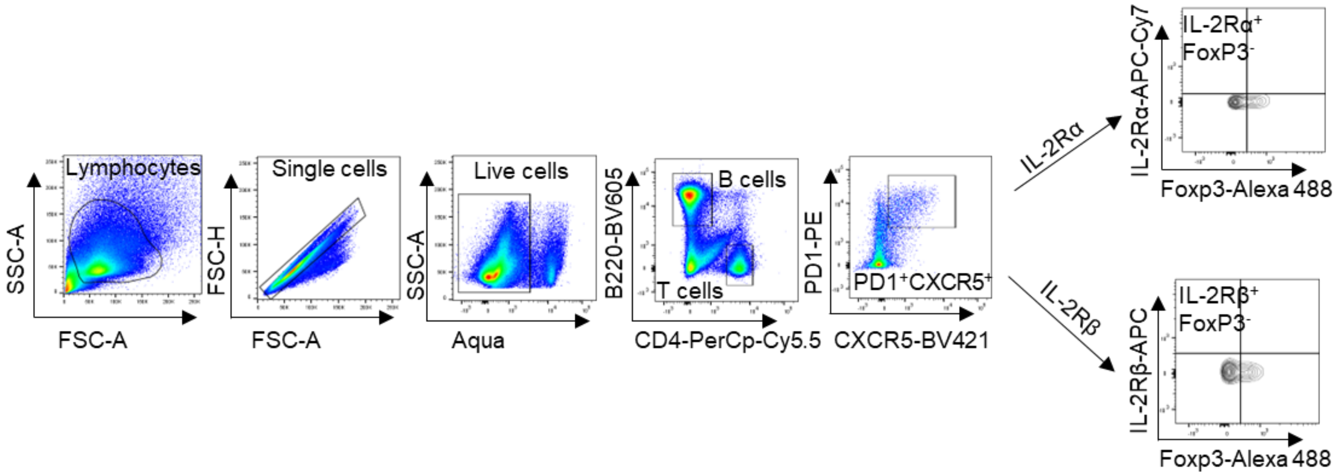

b **Gating strategy for pSTAT5⁺ of CD4⁺FoxP3⁻ T_{FH} cells**

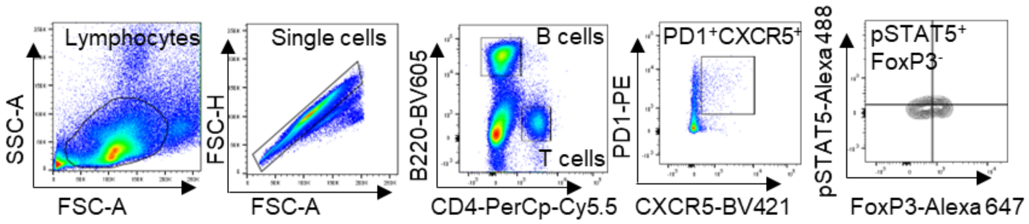

Supplemental Figure 6. (a) Gating strategy for the detection of IL-2Rα$^+$ or IL-2Rβ$^+$ cells on T_{FH} cells in splenocyte pools by FACS. Dead cells were excluded with LIVE/DEAD (Aqua) dye and cells were further stained with antibodies against CD4, B220, PD-1, CXCR5, FoxP3, IL-2Rα and IL-2 Rβ. IL-2Rα- and IL-2Rβ-expressing T_{FH} cells were detected within the CD4$^+$ cells as CXCR5^{hi}PD-1^{hi}Foxp3$^-$IL-2Rα$^+$ and CXCR5^{hi}PD-1^{hi}Foxp3$^-$IL-2Rβ$^+$ populations, respectively. **(b)** Gating strategy for the detection of p-STAT5$^+$ on T_{FH} cells in splenocyte pools via flow cytometry. The cells are stained with CD4, B220, PD-1, CXCR5, FoxP3, and p-STAT5$^+$ antibodies. p-STAT5$^+$ T_{FH} cells were detected within the CD4$^+$ cells as CXCR5^{hi}PD-1^{hi}Foxp3$^-$p-STAT5$^+$ population.

Supp. Fig. 7

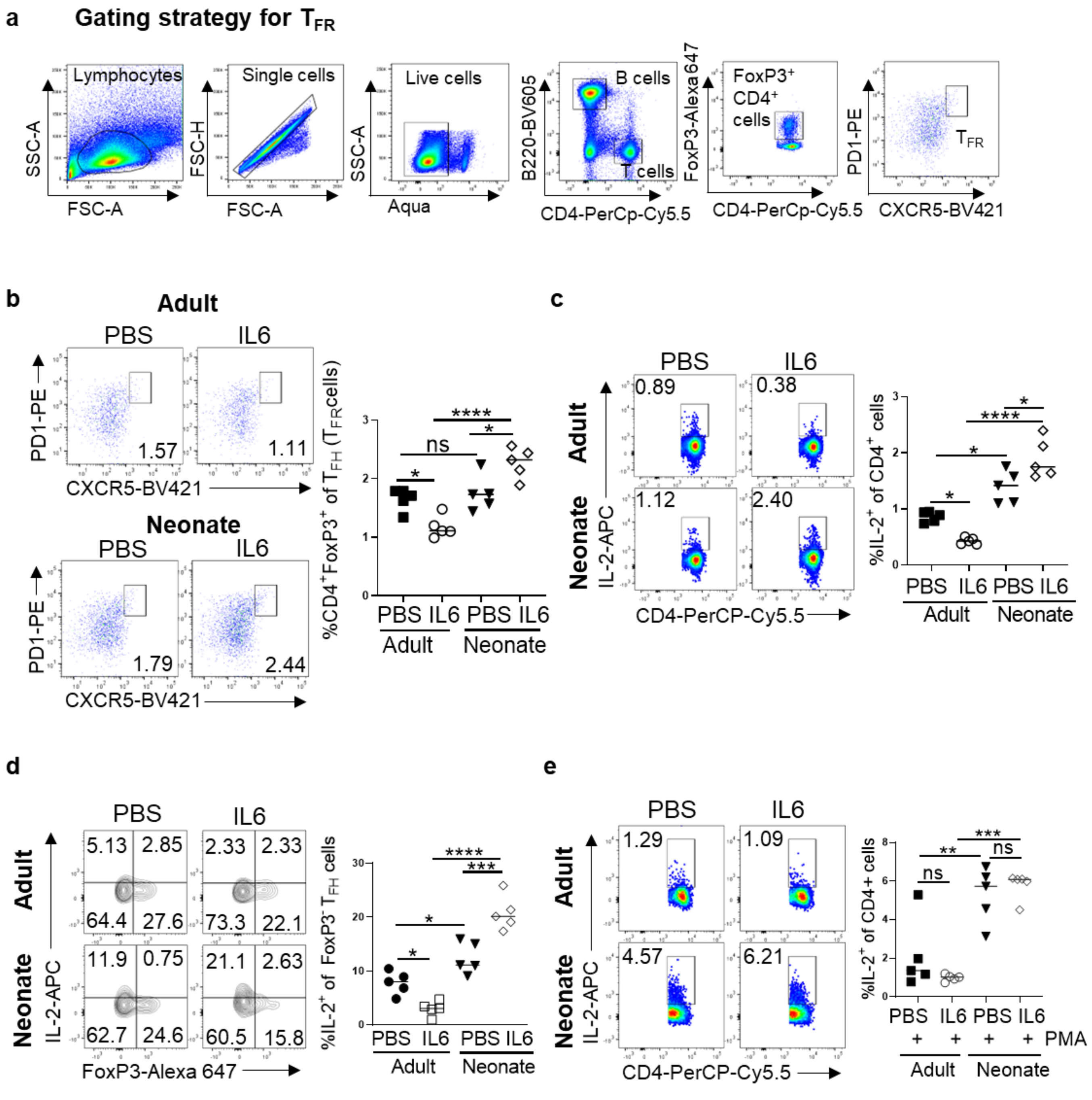

Supplemental Figure 7. Analysis of TFR cells and IL-2-producing CD4+ as well as TFH cells in IL-6 co-injected adult and neonatal mice. Adult (6- to 10-week-old) and neonatal (5- to 7-day-old) mice were immunized i.p. with PPS14-TT alone or PPS14-TT together

with IL-6 and splenocytes were analyzed by FACS. **(a)** Gating strategy for the detection of T_{FR} cells in splenocyte pools via flow cytometry. Dead cells were excluded with LIVE/DEAD (Aqua) dye and cells were further stained with antibodies against CD4, B220, PD-1, CXCR5 and FoxP3. T_{FR} were detected within the $CD4^+$ T cells as $FoxP3^+CXCR5^{hi}PD\text{-}1^{hi}$ population. **(b)** Representative dot plots show $CXCR5^{hi}PD\text{-}1^{hi}$ T_{FR} cells pre-gated on $CD4^+FoxP3^-$ population in immunized mice. Mean percentages of T_{FR} cells are plotted (n=5). **(c)** Representative dot plots depict the percentages of IL-2-expressing $CD4^+$ cells from immunized mice. Mean percentages of $IL\text{-}2^+$ among $CD4^+$ cells are plotted (n=5). **(d)** Representative counter plots depict the percentages of IL-2-expressing $FoxP3^+$ and $FoxP3^-$ cells pre-gated on T_{FH} ($CD4^+CXCR5^{hi}PD\text{-}1^{hi}$) population. Mean percentages of $IL\text{-}2^+$ cells among $FoxP3^-$ T_{FH} cells are plotted (n=5). **(e)** Splenocytes from immunized mice were in vitro stimulated with PMA/Ion for 4 hours and cells were analyzed by FACS. Representative dot plots depict the percentages of IL-2-expressing $CD4^+$ cells from stimulated splenocytes (n=5). Experiments were performed three times. One-Way ANOVA was used for all comparisons; data represented as mean +/- SEM are shown. P values <0.05 were considered statistically significant. *P<0.05, **P<0.01, ***P<0.001, ****P<0.0001 and ns (non-significant).

Supp. Fig. 8

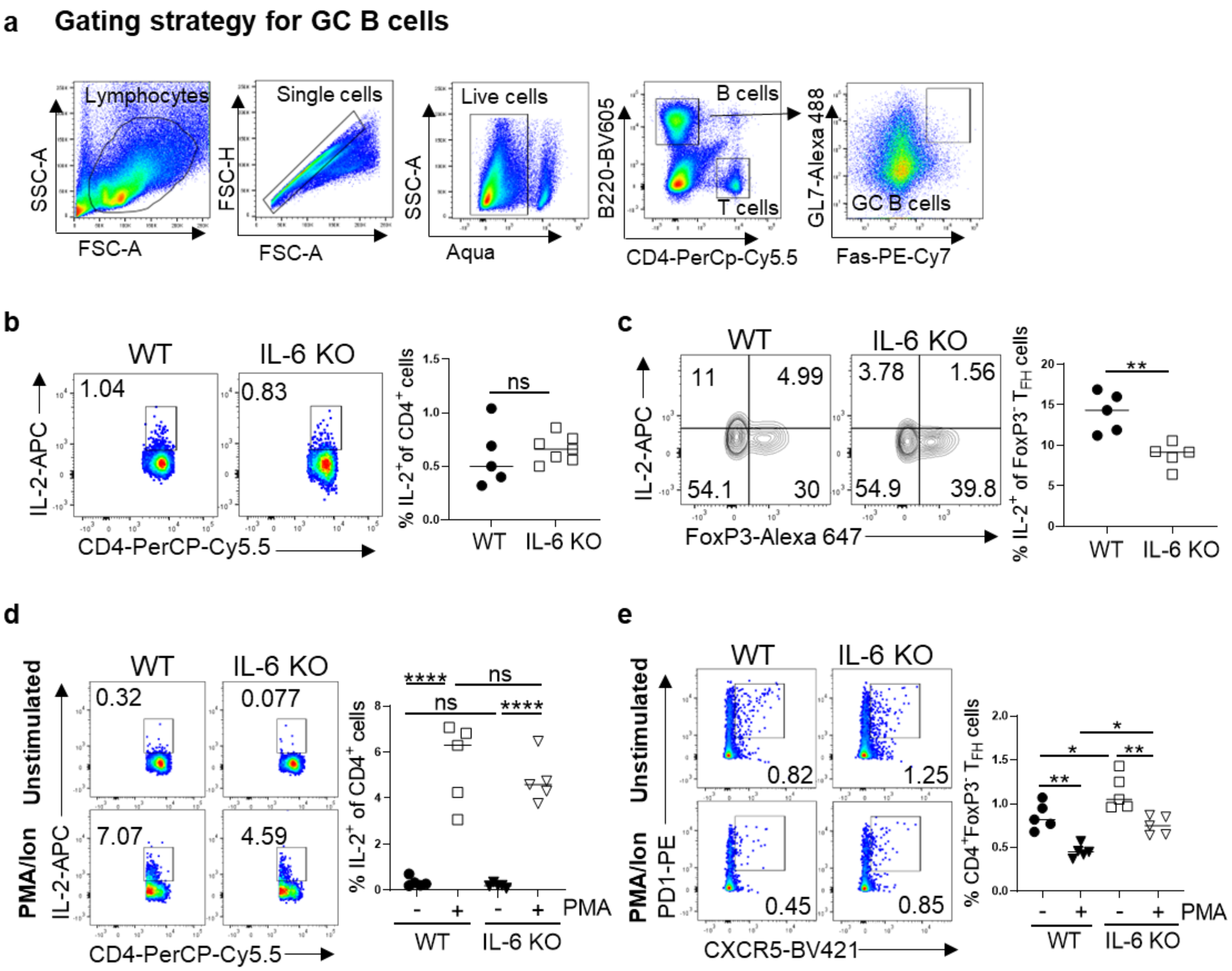

Supplemental Figure 8. (a) Gating strategy for the detection of GC B cells in splenocyte pools by FACS. Dead cells were excluded with LIVE/DEAD (Aqua) dye and cells were further stained with antibodies against CD4, B220, GL-7 and Fas. GC B cells were detected as B220^{+}GL-7^{+}Fas^{+} population. **(b-c)** Neonatal (5- to 7-day-old) wild-type (C57BL/6J) and IL-6 KO mice were immunized i.p. with PPS14-TT and splenocytes were analyzed by FACS. **(b)** Representative dot plots depict the percentages of IL-2-expressing CD4^{+} cells from immunized mice. Mean percentages of IL-2^{+} among CD4^{+}

cells are plotted (n=5). **(c)** Representative counter plots depict the percentages of IL-2-expressing FoxP3^{+} and FoxP3^{-} cells pre-gated on T_{FH} ($CD4^{+}CXCR5^{hi}PD\text{-}1^{hi}$) population. Mean percentages of IL-2^{+} cells among FoxP3^{-} T_{FH} cells are plotted (n=5). **(d-e)** Splenocytes from immunized mice were in vitro stimulated with PMA/Ion for 4 hours followed by intracellular staining for IL-2 on T_{FH} cells. **(d)** Representative dot plots depict the percentages of IL-2-expressing CD4^{+} cells from unstimulated and PMA/Ion splenocytes. Mean percentages of IL-2^{+} among CD4^{+} cells are plotted (n=5). **(e)** Representative counter plots depict the percentages of IL-2-expressing FoxP3^{+} and FoxP3^{-} cells pre-gated on T_{FH} ($CD4^{+}CXCR5^{hi}PD\text{-}1^{hi}$) population. Experiments were performed three to four times. Unpaired student's t-test and One-Way ANOVA was used for all comparisons; data represented as mean +/- SEM are shown. P values <0.05 were considered statistically significant. *P<0.05, **P<0.01, ****P<0.0001 and ns (non-significant).

Supp. Fig. 9

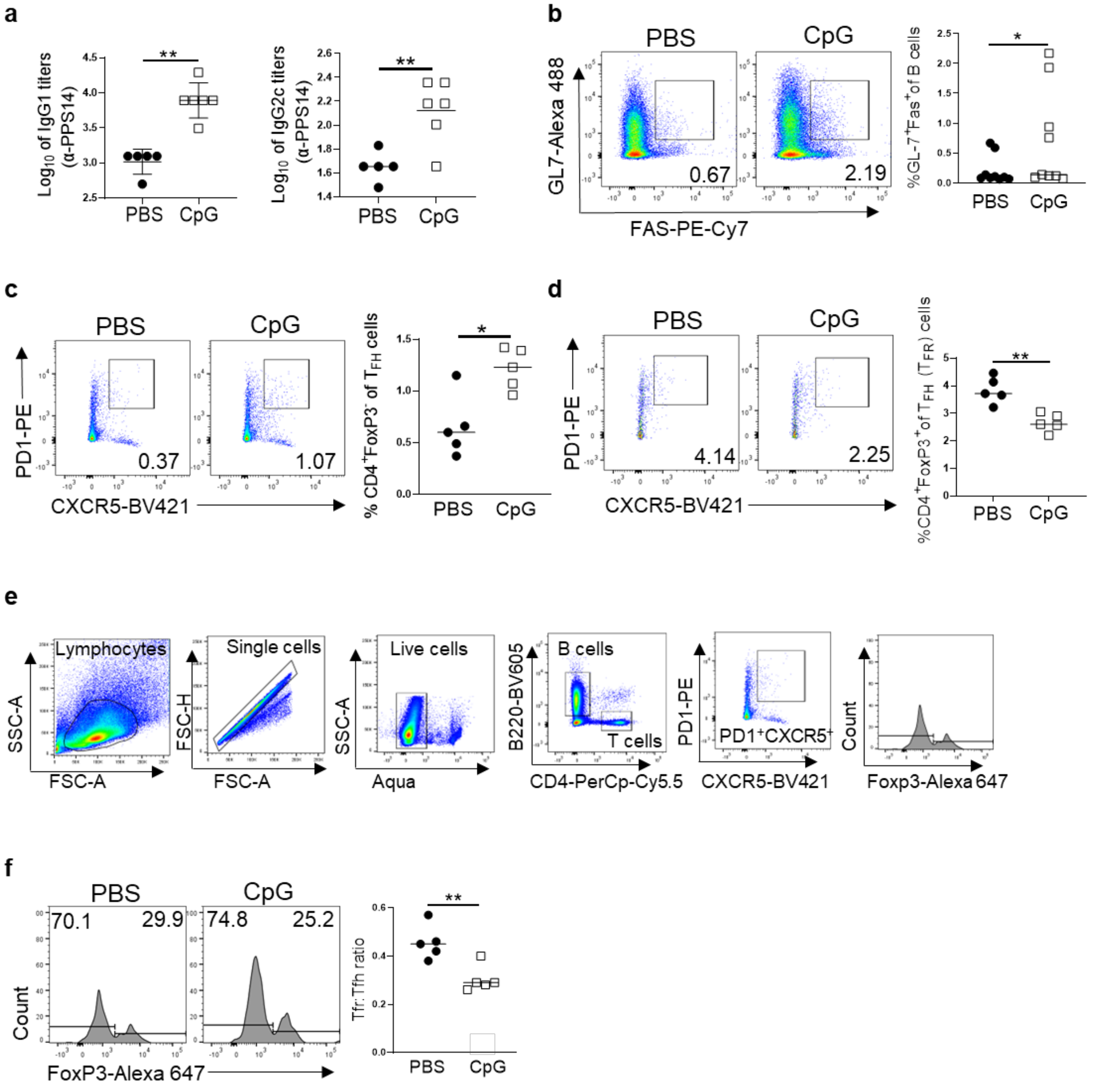

Supplemental Figure 9. Neonatal (5- to 7-day-old) C57BL/6J mice were immunized i.p. with PPS14-TT (PBS) or PPS14-TT + CpG (CpG) and splenocytes were analyzed by FACS at 7 dpi. **(a)** Serum anti-PPS14 IgG1 and anti-PPS14 IgG2c titers were determined by ELISA four weeks after immunization (n=5 PBS; n=6 CpG). **(b)** Representative dot plots depict the GC (GL7$^+$FAS$^+$) cells pre-gated on B220$^+$ cells.

Mean percentages of GC B cells are plotted (n=9). **(c)** Representative dot plots depict the T_{FH} ($CXCR5^{hi}PD\text{-}1^{hi}$) cells pre-gated on $CD4^{+}FoxP3^{-}$ population. Mean percentages of T_{FH} cells are plotted (n=5). **(d)** Representative dot plots depict T_{FR} ($CXCR5^{hi}PD\text{-}1^{hi}$) cells pre-gated on $CD4^{+}FoxP3^{+}$ cells. Mean percentages of T_{FR} cells are plotted (n=5). **(e)** Gating strategy for T_{FR}:T_{FH} ratio in splenocyte pools via flow cytometry. Dead cells were excluded with LIVE/DEAD (Aqua) dye and cells were further stained with antibodies against CD4, B220, PD-1, CXCR5 and FoxP3. T_{FH} cells were detected as $FoxP3^{-}$ and T_{FR} cells as $FoxP3^{+}$ cells pre-gated on $CXCR5^{hi}PD\text{-}1^{hi}$ cells, respectively. **(f)** Histograms represent the T_{FH} ($FoxP3^{-}$) and T_{FR} ($FoxP3^{+}$) populations on $CD4+CXCR5^{hi}PD\text{-}1^{hi}$ gated cells and the ratio of T_{FR} to T_{FH} cells (T_{FR}:T_{FH}) are plotted (n=5). Experiments were performed two to eight times. Unpaired student's t-test was used for all comparisons; data represented as mean +/- SEM are shown. P values <0.05 were considered statistically significant. *P<0.05, **P<0.01.

Supp. Fig. 10

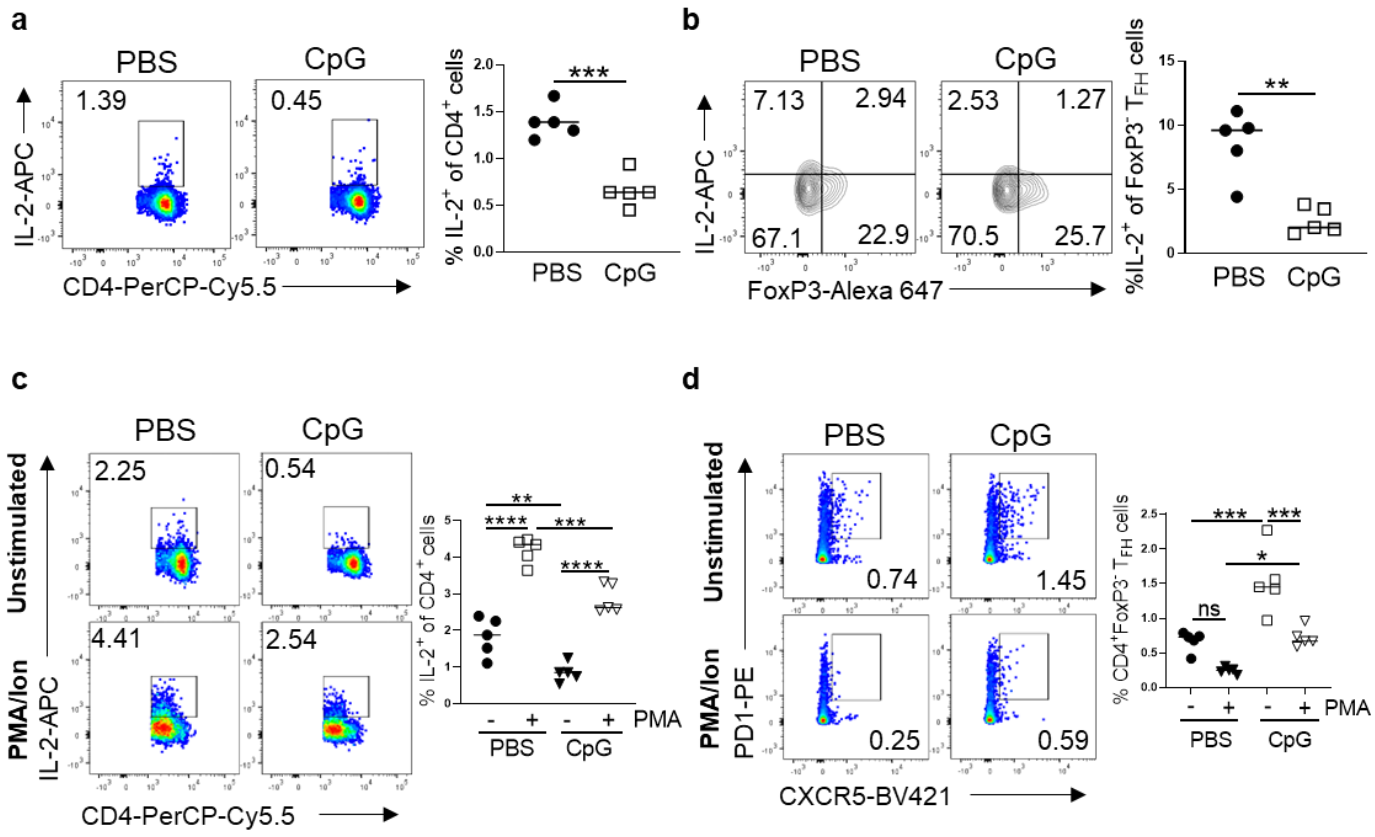

Supplemental Figure 10. Neonatal (5- to 7-day-old) C57BL/6J mice were immunized i.p. with PPS14-TT (PBS) or PPS14-TT + CpG (CpG) and splenocytes were analyzed by FACS at 7 dpi. **(a)** Representative dot plots depict the percentages of IL-2-expressing CD4$^+$ cells from immunized mice. Mean percentages of IL-2$^+$ among CD4$^+$ cells are plotted (n=5). **(b)** Representative counter plots depict the percentages of IL-2-expressing FoxP3$^+$ and FoxP3$^-$ cells pre-gated on T_{FH} (CD4$^+$CXCR5^{hi}PD-1^{hi}) population. Mean percentages of IL-2$^+$ cells among FoxP3$^-$ T_{FH} cells are plotted (n=5). **(c and d)** Splenocytes from immunized mice were in vitro stimulated with PMA/Ion for 4

hours followed by intracellular staining for IL-2 on T_{FH} cells. **(c)** Representative dot plots depict the percentages of IL-2-expressing $CD4^+$ cells from unstimulated and PMA/Ion splenocytes. Mean percentages of IL-2^+ among $CD4^+$ cells are plotted (n=5). **(d)** Representative counter plots depict the percentages of IL-2-expressing $FoxP3^+$ and $FoxP3^-$ cells pre-gated on T_{FH} ($CD4^+CXCR5^{hi}PD\text{-}1^{hi}$) population. Experiments were performed twice. Unpaired student's t-test and One-Way ANOVA was used for all comparisons; data represented as mean +/- SEM are shown. P values <0.05 were considered statistically significant. *P<0.05, **P<0.01, ***P<0.001, ****P<0.0001 and ns (non-significant).
