# Supplementary material for: IL6 suppresses vaccine responses in neonates by enhancing IL2 activity on T follicular helper cells
Source: NPJ Vaccines. 2023 Nov 8;8:173. doi: 10.1038/s41541-023-00764-1 (PMC10632457; doi:10.1038/s41541-023-00764-1)
Supplement: Supplementary file 2 — Reporting Summary [file 41541_2023_764_MOESM2_ESM.pdf]

Corresponding author(s): Mustafa Akkoyunlu
Last updated by author(s): Aug 24, 2023

Reporting Summary

Nature Portfolio wishes to improve the reproducibility of the work that we publish. This form provides structure for consistency and transparency in reporting. For further information on Nature Portfolio policies, see our Editorial Policies and the Editorial Policy Checklist.

Statistics

For all statistical analyses, confirm that the following items are present in the figure legend, table legend, main text, or Methods section.

n/a	Confirmed	
☐	☒	The exact sample size (n) for each experimental group/condition, given as a discrete number and unit of measurement
☐	☒	A statement on whether measurements were taken from distinct samples or whether the same sample was measured repeatedly
☐	☒	The statistical test(s) used AND whether they are one- or two-sided<br>*Only common tests should be described solely by name; describe more complex techniques in the Methods section.*
☒	☐	A description of all covariates tested
☒	☐	A description of any assumptions or corrections, such as tests of normality and adjustment for multiple comparisons
☐	☒	A full description of the statistical parameters including central tendency (e.g. means) or other basic estimates (e.g. regression coefficient) AND variation (e.g. standard deviation) or associated estimates of uncertainty (e.g. confidence intervals)
☐	☒	For null hypothesis testing, the test statistic (e.g. F, t, r) with confidence intervals, effect sizes, degrees of freedom and P value noted<br>*Give P values as exact values whenever suitable.*
☒	☐	For Bayesian analysis, information on the choice of priors and Markov chain Monte Carlo settings
☒	☐	For hierarchical and complex designs, identification of the appropriate level for tests and full reporting of outcomes
☒	☐	Estimates of effect sizes (e.g. Cohen's d, Pearson's r), indicating how they were calculated

Our web collection on statistics for biologists contains articles on many of the points above.

Software and code

Policy information about availability of computer code

Data collection	No custom algorithms or software was used in this manuscript.
Data analysis	Flow cytometry data were analyzed using FlowJo_v10.8.1 (BD, Ashland OR).<br>Statistical Analysis was performed using GraphPad Prism 9.

For manuscripts utilizing custom algorithms or software that are central to the research but not yet described in published literature, software must be made available to editors and reviewers. We strongly encourage code deposition in a community repository (e.g. GitHub). See the Nature Portfolio guidelines for submitting code & software for further information.

Data

Policy information about availability of data

All manuscripts must include a data availability statement. This statement should provide the following information, where applicable:

- Accession codes, unique identifiers, or web links for publicly available datasets
- A description of any restrictions on data availability
- For clinical datasets or third party data, please ensure that the statement adheres to our policy

All data generated or analyzed during this study are included in this published article (and its supplementary information files).

Research involving human participants, their data, or biological material

Policy information about studies with human participants or human data. See also policy information about sex, gender (identity/presentation), and sexual orientation and race, ethnicity and racism.

Reporting on sex and gender	*Use the terms sex (biological attribute) and gender (shaped by social and cultural circumstances) carefully in order to avoid confusing both terms. Indicate if findings apply to only one sex or gender; describe whether sex and gender were considered in study design; whether sex and/or gender was determined based on self-reporting or assigned and methods used. Provide in the source data disaggregated sex and gender data, where this information has been collected, and if consent has been obtained for sharing of individual-level data; provide overall numbers in this Reporting Summary. Please state if this information has not been collected. Report sex- and gender-based analyses where performed, justify reasons for lack of sex- and gender-based analysis.*
Reporting on race, ethnicity, or other socially relevant groupings	*Please specify the socially constructed or socially relevant categorization variable(s) used in your manuscript and explain why they were used. Please note that such variables should not be used as proxies for other socially constructed/relevant variables (for example, race or ethnicity should not be used as a proxy for socioeconomic status). Provide clear definitions of the relevant terms used, how they were provided (by the participants/respondents, the researchers, or third parties), and the method(s) used to classify people into the different categories (e.g. self-report, census or administrative data, social media data, etc.) Please provide details about how you controlled for confounding variables in your analyses.*
Population characteristics	*Describe the covariate-relevant population characteristics of the human research participants (e.g. age, genotypic information, past and current diagnosis and treatment categories). If you filled out the behavioural & social sciences study design questions and have nothing to add here, write "See above."*
Recruitment	*Describe how participants were recruited. Outline any potential self-selection bias or other biases that may be present and how these are likely to impact results.*
Ethics oversight	*Identify the organization(s) that approved the study protocol.*

Note that full information on the approval of the study protocol must also be provided in the manuscript.

Field-specific reporting

Please select the one below that is the best fit for your research. If you are not sure, read the appropriate sections before making your selection.

☒ Life sciences ☐ Behavioural & social sciences ☐ Ecological, evolutionary & environmental sciences

For a reference copy of the document with all sections, see nature.com/documents/nr-reporting-summary-flat.pdf

Life sciences study design

All studies must disclose on these points even when the disclosure is negative.

Sample size	Sample sizes were not predetermined based on certain statistical methods. The number of animals per group in our study (n=5) generated data that gave sufficient statistics for the effect sizes of interest.
Data exclusions	No data are excluded from consideration.
Replication	For biological replicates, five animals were assigned to each group. Each experiment was done at least twice.
Randomization	Animals were randomly assigned to different groups in the study.
Blinding	Investigators were not blinded in this study. This is an animal study and blinding is not typically used in this situation.

Reporting for specific materials, systems and methods

We require information from authors about some types of materials, experimental systems and methods used in many studies. Here, indicate whether each material, system or method listed is relevant to your study. If you are not sure if a list item applies to your research, read the appropriate section before selecting a response.

Materials & experimental systems

n/a	Involved in the study
☐	☒ Antibodies
☒	☐ Eukaryotic cell lines
☒	☐ Palaeontology and archaeology
☐	☒ Animals and other organisms
☒	☐ Clinical data
☒	☐ Dual use research of concern
☒	☐ Plants

Methods

n/a	Involved in the study
☒	☐ ChIP-seq
☐	☒ Flow cytometry
☒	☐ MRI-based neuroimaging

Antibodies

Antibodies used	α-CD4 (BD Biosciences, GK1.55), α-B220 (BioLegend, RA3-6B2), α-PD-1 (BD Biosciences, J43 or BioLegend, 29F.1A12), α-CXCR5 biotin (BD Biosciences, 2G8), α-GL7 (BD Biosciences, GL-7), α-CD95 (BD Biosciences, J02), α-IL-2Rα (BioLegend, PC61), α-IL-2Rβ (BioLegend, TM-β1), α-CD19 (BioLegend, 6D5), α-CD3 (BioLegend, 17A2), α-CD11c (BioLegend, N418), Streptavidin-BV-421 (BD Bioscience), α-FoxP3 (BD Biosciences, MF23 or BioLegend, 150D), α-IL-2 (BD Biosciences, JES6-5H4) or α-IL-6 (BioLegend, MP5-20F3), p-STAT5 (BD Bioscience, pY694, clone 47) and p-STAT3 (BD Biosciences, pY705, Clone 13A3-1).
Validation	Validation was performed by vendors. All the antibodies were used as per the manufacturer's recommendation.

Animals and other research organisms

Policy information about studies involving animals; ARRIVE guidelines recommended for reporting animal research, and Sex and Gender in Research

Laboratory animals	6- to 10- week (adult) old C57BL/6J were purchased from Jackson labs. Breeding pairs were set up in-house to obtain neonatal mice. IL-6 KO breeding pairs were purchased from Jackson labs.
Wild animals	Wild animals were not used in this study
Reporting on sex	For Adult mice, females were used. 5- to 7- day old mice were used as neonates for which no sex determination was done.
Field-collected samples	Field-collected samples were not used in this study
Ethics oversight	Animal protocol was approved by FDA's Institutional Animal Care and Use Committee (Protocol 2017-48)

Note that full information on the approval of the study protocol must also be provided in the manuscript.

Flow Cytometry

Plots

Confirm that:

☒ The axis labels state the marker and fluorochrome used (e.g. CD4-FITC).

☒ The axis scales are clearly visible. Include numbers along axes only for bottom left plot of group (a 'group' is an analysis of identical markers).

☒ All plots are contour plots with outliers or pseudocolor plots.

☒ A numerical value for number of cells or percentage (with statistics) is provided.

Methodology

Sample preparation	Single cell suspensions prepared from spleen tissue of mice were used for flow cytometric analysis.
Instrument	Fortessa, BD
Software	FlowJo_v10.8.1
Cell population abundance	No cell sorting was performed in this study
Gating strategy	Splenocytes were gated by size (SSC-A vs FSC-A) gating and singlet (FSC-A vs FSC-H) gating.

☒ Tick this box to confirm that a figure exemplifying the gating strategy is provided in the Supplementary Information.
